# Effects of telephone-based health coaching on patient-reported outcomes and health behavior change: A randomized controlled trial

**DOI:** 10.1371/journal.pone.0236861

**Published:** 2020-09-22

**Authors:** Sarah Dwinger, Farhad Rezvani, Levente Kriston, Lutz Herbarth, Martin Härter, Jörg Dirmaier

**Affiliations:** 1 Department of Medical Psychology, University Medical Center Hamburg-Eppendorf, Hamburg, Germany; 2 Kaufmännische Krankenkasse, Statutory Health Insurance, Hannover, Germany; Universidad Miguel Hernandez de Elche, SPAIN

## Abstract

**Objective:**

Telephone based health coaching (TBHC) seems to be a promising approach to foster self-management in patients with chronic conditions. The aim of this study was to evaluate the effectiveness of a TBHC on patient-reported outcomes and health behavior for people living with chronic conditions in Germany.

**Methods:**

Patients insured at a statutory health insurance were randomized to an intervention group (IG; TBHC) and a control group (CG; usual care), using a stratified random allocation before giving informed consent (Zelen’s single-consent design). The TBHC was based on motivational interviewing, goal setting, and shared decision-making and carried out by trained nurses. All outcomes were assessed yearly for three years. We used mixed effects models utilizing all available data in a modified intention-to-treat sample for the main analysis. Participants and study centers were included as random effects. All models were adjusted for age, education and campaign affiliation.

**Results:**

Of the 10,815 invited patients, 4,283 returned their questionnaires at baseline. The mean age was 67.23 years (SD = 9.3); 55.5% were female. According to the model, TBHC was statistically significant superior to CG regarding 6 of 19 outcomes: physical activity in hours per week (p = .030) and in metabolic rate per week (p = .048), BMI (p = .009) (although mainly at baseline), measuring blood pressure (p< .001), patient activation (p< .001), and health literacy (p< .001). Regarding stages of change (p = .005), the IG group also showed statistically different results than the CG group, however the conclusion remains inconclusive. Within-group contrasts indicating changes from baseline to follow-ups and significant between-group comparisons regarding these changes supported the findings. Standardized effect sizes were small. TBHC did not show any effect on mental QoL, health status, alcohol, smoking, adherence, measuring blood sugar, foot monitoring, anxiety, depression and distress. Campaign-specific subgroup effects were detected for ‘foot monitoring by a physician’ and ‘blood sugar measurement’.

**Conclusion:**

TBHC interventions might have small effects on some patient reported and behavioral outcomes.

**Practice implications:**

Future research should focus on analyzing which intervention components are effective and who profits most from TBHC interventions.

**Registration:**

German Clinical Trials Register (Deutsches Register Klinischer Studien; DRKS): DRKS00000584

## 1. Introduction

Due to better medical treatment and changes in demographics, an ageing population will result in increasing numbers of people living with chronic conditions. In 2010, 15% of Europe’s population was older than 65 years; prognoses expect an increase to 25% in 2050 [1]. In Europe, chronic conditions account for 80% of the mortality; particularly diseases of the circulatory system account for nearly 50% [1, 2]. In addition to those patients affected by one chronic condition (24.3%), the proportion of multimorbid patients is very high: 13.8% had two and 11.7% had more than three chronic conditions [3], resulting in a reduction in life expectancy by about 1.8 years with each additional chronic condition for a 67-year old individual [4]. Besides accounting for most part of the health care expenditures and lost work productivity [5, 6], chronic conditions also have a large impact on the individual living with it. Cardiovascular diseases for example are responsible for the most lost “disability-adjusted life-years” (DALYs) in low- and middle-income countries in Europe, and the third most lost DALYs in high-income countries [1, 7].

However, an existing chronic condition and its impact on a person’s life may be modifiable in several ways, such as adopting better health behaviors and a better self-management. Studies show that the consumption of alcohol and tobacco, as well as high blood pressure are the three most important risk factors predicting a higher disease burden [1]. Therefore, current treatment guidelines, like the NICE guideline for managing diabetes [8], as well as the disease management program (DMP) guidelines for diabetes, breast cancer, asthma, and coronary heart disease in Germany [9], include self-management trainings and lifestyle change as a part of the medical treatment of many chronic conditions. Also in the US, the enhancement of self-management abilities and patient empowerment are major goals, as stated in the “Strategic Framework on Multiple Chronic Conditions” of the US Department of Health and Human Services [10].

Meta-analyses show that self-management interventions can improve quality of life (QoL) as well as disease-specific outcomes and decrease health care costs [11–13]. Besides self-management courses in group settings [14], one promising approach to improve self- and disease-management in people living with chronic conditions is telephone-based health coaching (TBHC), which is more accessible to people living in rural areas or having limited mobility. However, evidence regarding the effectiveness of TBHC is still inconclusive: Reviews conclude that TBHC may have beneficial effects on some clinical, behavioral, and psychosocial outcomes [10, 15, 16], but the heterogeneity, for example in duration and delivery method (e.g. calls, video calls, short messages, automated messages), of the TBHC interventions included, as well as the narrative review methods, make it difficult to draw clear conclusions.

Studies reporting the effect on patient-reported outcomes, like QoL, mental status, and distress are rare and have not been thoroughly summed up. A systematic review about the effectiveness of TBHC for chronic conditions found that results regarding psychosocial outcomes were quite inconclusive [15]. Most studies reported no effect of TBHC on “overall QoL” [17–22], but there were effects on “physical QoL” [19, 22–24]. Although not the focus of most of the studies, the effects of TBHC on anxiety of people living with chronic conditions have been quite positive [25, 26]. On the contrary, effects of TBHC on depression were mixed, but more likely to show no effect in favor of TBHC compared to controls [17, 19, 25, 27, 28].

Change of health behavior is a key focus in many TBHC interventions. Nevertheless, a review of current research we conducted showed no difference between a TBHC intervention group (IG) and a control group (CG) regarding most health behavior outcomes, like exercise [23, 29–37], self-monitoring [29, 34, 35, 38], alcohol [28, 39, 40], and smoking [17, 25, 27, 28, 32, 41]. Yet, there are promising results in favor of TBHC for diet change [24, 25, 31, 33–35, 38, 42].

Most existing studies were conducted in the United Stated, Australia, and the United Kingdom, which leads to different health systems and quite diverse populations, especially in countries with huge rural areas. There is limited data on TBHC in Europe; in particular there is no study on TBHC outcomes in Germany besides the present study [43, 44] and its pilot study [45]. The evaluation of health economic outcomes of this study showed no effect of the TBHC on the time until and probability of rehospitalization, number of daily defined medication doses (medication), as well as frequency and duration of inability to work. Nevertheless, there was a reduction of hospitalization in participants with heart failure, and a reduction of mortality in participants with chronic somatic conditions [44].

### 1.1 Objectives

The aim of this study was to evaluate the effects of a TBHC for people living with chronic conditions on 1) QoL, 2) health behaviors, (e.g. treatment or medication adherence, smoking, and alcohol consumption), as well as 3) psychosocial outcomes, (e.g. depression, anxiety, health literacy, patient activation and stages of change) compared to a CG of patients receiving usual care.

## 2. Materials and methods

The methods, including design, randomization process and all measures have been described elsewhere [43, 44]. The primary outcome of this study was “time from enrolment until hospital readmission within two years” which was assessed using routine data of the statutory health insurance data set. Together with further health economic outcomes (e.g. health related costs, inability to work, and mortality) the corresponding results are presented and discussed elsewhere [44]. Here we report findings on patient-reported secondary outcomes of the study.

### 2.1 Study design

In this 4-year (June 2010 to October 2014) prospective, pragmatic randomized controlled trial (RCT) we compared participants receiving a TBHC intervention with patients in usual care. Using Zelen’s single-consent design, patients were randomized into IG or CG before giving informed consent [46] due to ethical reasons within the statutory health insurance. If patients declined TBHC, they received usual care. In addition to baseline measure (T_0_) there were three follow-up measures at 12 months (T_1_), 24 months (T_2_), and 36 months (T_3_). A study protocol reporting rationale, study design and statistical analysis procedures has been published a priori [43].

The study complied with the Helsinki Declaration 2008. The ethics approval was granted by the Hamburg Medical Chamber Ethics Committee on the 12.05.2011 (process number: PV3567). The study was registered in the German Clinical Trials Register (DRKS00000584). The trial was registered nine months late due to unexpected delays in the course of contract negotiations with the funding institution (Kaufmännische Krankenkasse Hannover: KKH) and resulting problems with timely recruitment of scientific staff. Nevertheless, registration was completed before any data were analyzed. The authors confirm that all ongoing and related trials for this intervention are registered. The consort checklist can be found in S1 Table.

### 2.2 Sample

The sample consisted of patients insured at the statutory health insurance KKH, which met the inclusion criteria within the recruitment period.

#### 2.2.1 Inclusion criteria and exclusion criteria

Study participants were adults (≥ 18 years), insured at KKH, and diagnosed with at least one chronic condition. Based on the diagnosed condition, eligible persons were grouped into different campaigns: The “chronic campaign” was utilized for type 2 diabetes, hypertension, and coronary artery disease; the “heart failure campaign” for heart failure patients, and the “mental health campaign” for chronic depression and schizophrenia. For the “chronic campaign” there were two different identification ways: For ‘chronic campaign 1’ patients were identified by a previous hospital stay, for ‘chronic campaign 2’ patients were identified by a risk score. If an insurant has more than one chronic conditions diagnosis, he is grouped in the most specific campaign in following order: “mental health campaign”, “heart failure campaign”, “chronic campaign”. Insurees were excluded if they were not able to understand German, had hearing impairment, or were not able to use a phone [43].

#### 2.2.2 Procedures

Successive recruitment took place between June 2010 and October 2011, with the follow-ups exactly 1, 2 and 3 years later. The members of the randomized IG received an invitation to take part in the TBHC and an acquisition call by the health insurance nurses. After sending back the informed consent for taking part in the intervention to the health insurance, they were included in the study as “TBHC participants”. In case they did not send back the required confirmation they were grouped as “TBHC decliners”. The randomized CG did not receive an invitation. To avoid bias also decliners received questionnaires, but due to economic reasons, decliners group and CG were randomly limited to 3,000 patients. All included patients received the consent form for the study with the baseline questionnaire, which had to be sent back to the research institute for taking part in the study. Patient reported data were collected by questionnaires sent to the insured persons’ home by the statutory health insurance.

#### 2.2.3 Randomization

All eligible persons were blindly randomized by a computer algorithm at the statutory health insurance’s headquarters to either IG or CG. Inclusion and randomization process took place gradually over a period of 14 months. We used a stratified random allocation design based on sociodemographic variables available in routine data. The randomization process was carried out by the statutory health insurance.

#### 2.2.4 Blinding

Blinding of study participants and coaches was not possible, as the coaching is provided one-to-one. However, the coaches did not know who did answer the questionnaires and who did not. The questionnaires were pseudonomyzed, to enable an aggregation of data from different sources.

#### 2.2.5 Sample size calculation

The a priori power calculation showed that an overall minimum of 1,670 patients were needed at T_2_ to be able to detect a small standardized mean difference (Cohen’s d of 0.2) in group comparisons in order to achieve a power of at least 95% at a type I error rate of α = 5% in a two-sided test accounting for the unbalanced group allocation [47]. Based on experiences in the pilot study showing low response and high drop-out rates [45], we targeted to invite 12,000 patients to participate, but achieved 10,815.

### 2.3 Intervention

The intervention is described following the TIDieR checklist [48] in S2 Table. The TBHC concept was originally developed by Health Dialog Inc. [49, 50], adapted to the German health care system and, subsequently, widely implemented by the health insurance KKH. A pilot study indicated that the intervention was well accepted by the participants [45]. Important components and counselling strategies were motivational interviewing (MI) to increase willingness to change and confidence to implement changed behaviors in daily life, individual and collaborative goal setting, and shared decision-making (SDM) [49, 50]. SDM focused on shared information on advantages and disadvantages of health behaviors and a joint decision. The set goals were recorded by the coach and followed-up in the upcoming calls. The intervention was tailored to important chronic conditions that require similar self-management strategies in the three campaigns “chronic campaign”, “heart failure campaign”, and “mental health campaign”. Although patients were identified for the ‘chronic campaign’ in two different ways, the intervention was the same.

The intervention was conducted by 20 nurses and one ecotrophologist located in two call centers (Munich and Halle/Saale). The coaches were trained in TBHC with MI and SDM components by experts directly trained by Health Dialog. They were supervised two to four times per year by two experienced supervisors from the project group (MH,IBB).

The minimum call frequency was defined as one telephone contact every six weeks with a maximum intervention duration of one year. Specific intervention manuals for the coaches regarding different situations (e.g. for smoking cessation), available topics, and accessible information materials provided support for the coaches. Also, the coaches were assisted by an online health platform (www.netdoktor.de) providing evidence-based and up-to-date health information. NetDoktor is a health portal written and edited by health professionals, certified by HONcode (www.hon.ch) and related to the afgis criteria (www.afgis.de), two quality certifications for reliable online health information. Data on the coaching process, individual goal setting, medication, and clinical parameters (e.g. Hb_A1c_ and blood pressure) were recorded by the coach in an electronic documentation system. Written patient information for specific conditions, medication plans, and weight-control tables could be sent to the TBHC participants. Additionally, participants in the heart failure campaign got a booster call, in which the coaches checked whether participants maintained health behaviors (e.g., weighing and medication adherence).

The CG received no coaching.

### 2.4 Measures

We assessed changes in QoL with the subscales “mental QoL” and “physical QoL” of the “Short Form 12 Health Survey” (SF-12) [51] and the health status with the visual analogue scale of the “EuroQol- 5 Dimension” (EQ-5D) [52].

**Health behaviors** (alcohol consumption, medication adherence, exercise) were assessed with the “Alcohol Consumption Questions of the Alcohol Use Disorders Identification Test” (AUDIT-C) [53], the “Medication Adherence Report Scale” (MARS-D) [54], and the “Freiburg Questionnaire for Physical Activity” (FFKA) [55]. The FFKA calculates the activity in hours per week and metabolic rate per week. We used self-developed, ordinally scaled instruments for the assessment of the rate of measuring blood pressure (1 = not until now, 2 = not regularly, 3 = weekly, 4 = mostly once a day, 5 = twice a day or more), measuring blood sugar (1 = not until now, 2 = not regularly, 3 = mostly once a day in the morning, 4 = twice a day or more when eating), foot monitoring by themselves (1 = not until now, 2 = not regularly, 3 = once a week, 4 = daily), foot monitoring by their physician (1 = unnecessary, 2 = once in the last year, 3 = twice or more in the last year).

Other **psychosocial outcomes** included patient activation with the German version of the “Patient Activation Measure” (PAM) [56], health literacy with the “Functional Communicative Critical Health Literacy” (FCCHL) [57], and the process of behavior change with an adaptation of the “Stages of Change across 10 Health Risk Behaviors for older Adults” (SOC) [58]. Changes in depression and anxiety were assessed with the “Hospital Anxiety and Depression Scale” (HADS) [59]. Additionally, we assessed socio-demographic factors like age, nationality, sex, marital status, number of children, net income, years of school, level of education and occupation, as well as clinical parameters with self-developed, ordinally scaled items. All outcomes and times of assessment can be found in Table 1.

**Table 1:** Outcomes, measures and times of assessment.

Outcome	Measure	Score
**Quality of life**		
Mental QoL	SF-12 Mental subscale	0–100, Mean = 50 (SD = 10)
Physical QoL	SF-12 Physical subscale	0–100, Mean = 50 (SD = 10)
Health status	EQ-5D-Visual Analog Scale	0–100
**Health behaviors**		
Alcohol consumption	AUDIT-C	0–12
Medication adherence	MARS-D	5–25
Smoking	Self-developed	Yes / No
Measuring blood pressure	Self-developed	Ordinal scale
Measuring blood sugar	Self-developed	Ordinal scale
Foot monitoring self	Self-developed	Ordinal scale
Foot monitoring by physician	Self-developed	Ordinal scale
Physical activity	FFKA	Hours per week, metabolic rate per week
Body Mass Index (BMI)	Self-reported (height, weight)	
**Clinical parameters**		
Blood pressure	Self-developed	Ordinal scale
Hb_A1c_	Self-developed	Ordinal scale
Cholesterol	Self-developed	Ordinal scale
NYHA status	Self-developed	Ordinal scale
**Psychosocial outcomes**		
Patient activation	PAM	12–52
Stages of change	SOC	9–45
Health literacy	FCCHL	14–56
Anxiety	HADS-A anxiety subscale	0–21
Depression	HADS-D depression subscale	0–21
Total score	HADS-T distress; total score	0–42

QoL = quality of life; SF-12 = Short Form 12 Health Survey; EQ-5D = EuroQol- 5 Dimension; AUDIT-C = Alcohol Use Disorders Identification Test; MARS-D = Medication Adherence Report Scale; FFKA = Freiburg Questionnaire for Physical Activity; PAM = Patient Activation Measure; SOC = Stages of Change; FCCHL = Functional Communicative Critical Health Literacy; HADS = Hospital Anxiety and Depression Scale; all outcomes were assessed atT_0_, T_1_, T_2_, T_3_; T_0_ = Baseline; T_1_ = 12 months after baseline; T_2_ = 24 months after baseline; T_3_ = 36 months after baseline.

### 2.5 Statistical methods

We applied three different analyses to minimize participation bias. We followed an intention-to-treat approach including available data from all patients randomized to IG (TBHC participants and TBHC decliners) in one analysis to avoid bias (intention-to-treat 1, ITT-1). Thus, as the majority of the study participants invited to the IG declined participation in the allocated intervention, we ran two additional analyses: one comparing the TBHC participants only (i.e. removing decliners) with the CG (intention-to-treat 2, ITT-2) and finally, in the as-treated (AT) analysis we compared TBHC participants with a minimum of 5 calls to the CG (S1 Fig). We defined the ITT-2 analysis as our main outcome. Decliners were not added to the CG group to prevent a larger bias in ITT-2 and AT.

Chi-square tests (for categorical outcomes) and ANOVA tests (for dimensional outcomes) were used to compare groups at baseline.

Mixed models with maximum likelihood estimation were used to test the impact of health coaching on the course of outcomes from baseline across the three follow-ups compared to routine care. In all models, intervention group (IG and CG), time (t_0_, t_1_, t_2_, t_3_) and the interaction between group and time (‘time x group’) were set as fixed effects. Participants and study centers were included as random effects. Due to group differences, models were adjusted for the campaign they were in (“chronic campaign” (‘chronic campaign 1’, ‘chronic campaign 2’), “heart failure campaign”, “mental health campaign”) and some sociodemographic variables (ITT-1: education; ITT-2: education, age; AT: education). In contrast to the health economic publication [44] we decided to subdivide the chronic campaign into its two smaller subgroups, which differed regarding their inclusion criteria, to avoid a possible bias and detect potential group differences.

The effect of health coaching was estimated based on the interaction between group and time. For group comparisons we calculated standardized between-group effect sizes (Cohen’s d) by dividing the estimated marginal means difference (EMM difference) of the groups by the observed standard deviation of the CG group. Post hoc interaction contrasts between group and time, i.e. the difference between the two groups in the amount of change from baseline (t_0_) to post-intervention measurements (t_1_, t_2_, t_3_), indicative of treatment effects, were determined.

Additionally, we tested whether campaigns moderate the effectiveness of the health coaching intervention, including the interaction between group, time and campaign (along with all lower level interactions) as a fixed effect in the analysis.

Across all tests, we considered results with p < 0.05 as statistically significant. Mixed model analyses were performed with R version 9.2 [60] using the lmer command from the lme4 package. Differences between the groups at baseline, imputation of missing values, as well as observed values were conducted with IBM SPSS 23 [61].

Although stated otherwise in the study protocol [43], we preferred the mixed model approach over the ANCOVA, as it utilizes data across all measurement points simultaneously and is more robust against missing values. Also, we did not adjust for multiple comparisons given the explorative nature of this study. As the Bonferroni correction increases the type II error in favor for decreasing type I error, we decided to follow Perneger (1998) to describe the results openly and discuss them carefully [62].

### 2.6 Missing values

In order to calculate sum scores, it was necessary to impute missing values on item level. First, to check whether the missing values were missing at random, we applied Littles MCAR test [63]. It showed that missing values could not be considered to be missing completely at random at all times (t_0_, t_1_, t_2_, t_3_). Therefore, we decided to use the expectation-maximization algorithm for imputing missing values on single item level across each scale at each time point, as it is assumed to be unbiased and efficient even though missing mechanisms may be unclear [64]. Also, we decided to impute values just for those patients that provided more than 70% valid responses in accordance with Wirtz (2004) [65]. If there were data missing due to lost to follow-up, there was no imputation done since mixed model analyses provide unbiased estimates under the assumption that data are missing at random conditional on the variables in the model [66]. Therefore, we did not use last observation carried forward as initially planned [43].

## 3.Results

### 3.1 Participant flow

10,815 patients were eligible for TBHC, with 7,582 allocated to IG (70%) and 3,233 allocated to CG (30%). Unfortunately, there are no data on how many people were excluded based on which eligibility criterion. 3,229 people (42.6%) of the IG received the allocated intervention (TBHC participants), whereas 4,353 (57.4%) declined participation (TBHC decliners). 1,767 (54.7%) of the TBHC participants provided baseline data, 45.3% did not respond. 1,353 (31.1%) of the TBHC decliners were randomly excluded and did not receive a questionnaire, of the remaining N = 3,000, 1,294 (43.1%) sent back the baseline questionnaire and 1,706 (56.9%) did not respond. Of the CG 233 patients (7.2%) were randomly excluded due to economic reasons, with 1,222 (40.7%) of the remaining N = 3,000 providing baseline data, and 2,011 (67.0%) not sending back the questionnaire. At t_2_, of those providing baseline data, 1,134 (64.2%) of the TBHC participants, 685 (52.9%) of the TBHC decliners, and 722 patients (59.1%) of the CG also provided t_2_ data. Three years after randomization 932 (52.7%) of the TBHC participants, 576 (44.5%) of the decliners and 608 (49.8%) of the CG provided baseline and t_3_ follow-up data.

**Fig 1:**
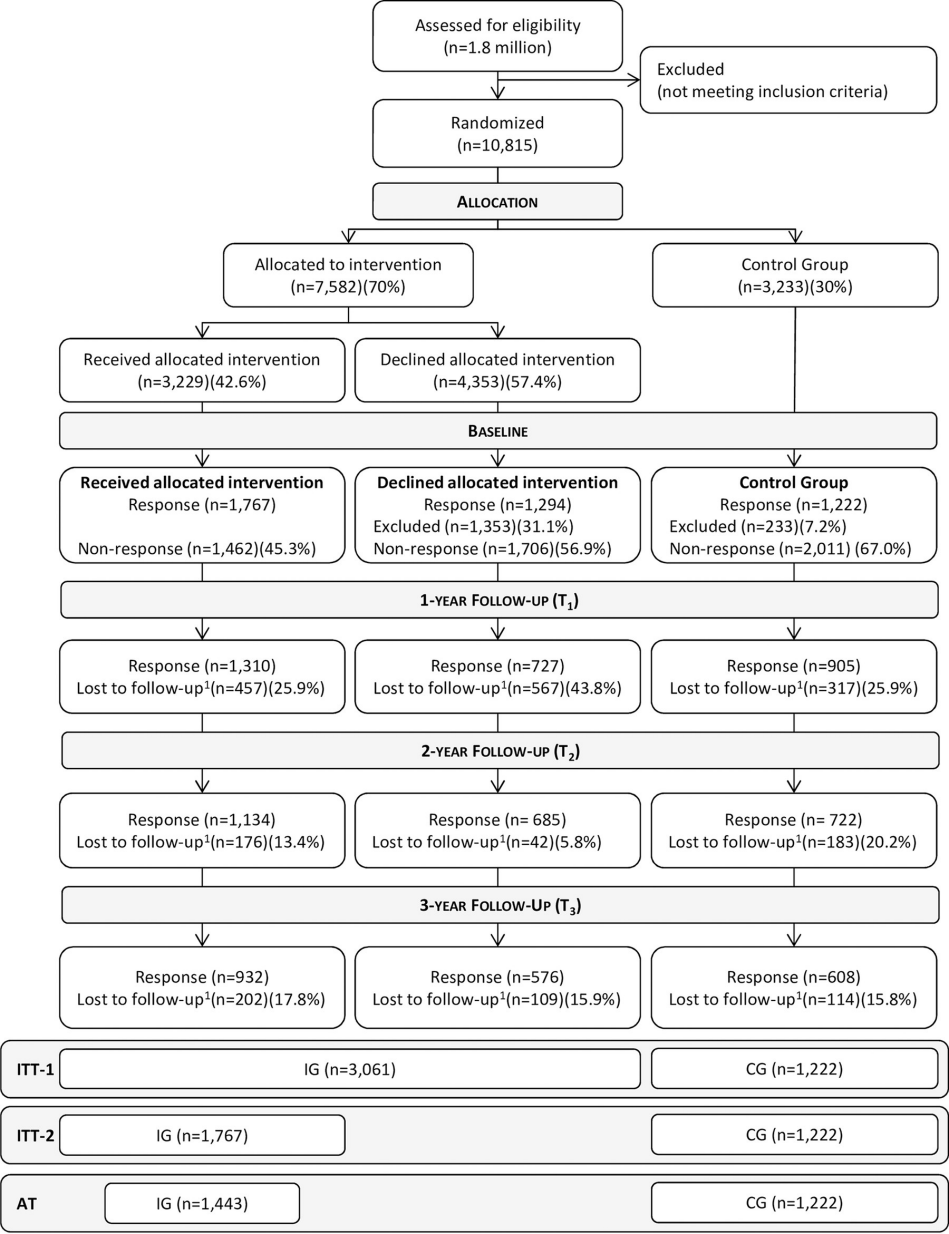
CONSORT participant flow chart. ^1^ = due to non-response / can include death; ITT-1 = intention-to-treat analysis 1 (n = 4,283); ITT-2 = intention-to-treat analysis 2 (n = 2,989); AT = as treated analysis (n = 2,665).

As the analysis will include all patients that responded at baseline, the sample will be as follows: 4,283 patients in ITT-1 (IG: N = 3,061; CG: N = 1,222), 2,989 patients in ITT-2 (IG: N = 1,767; CG: N = 1,222), and 2,665 patients in AT (IG: N = 1,443; CG: N = 1,222). The detailed participant flow is provided in Fig 1.

### 3.2 Baseline characteristics

Of the 4,283 patients that returned their questionnaires at baseline, 41.3% belonged to the participants, 30.2% declined participation, and 28.5% belonged to the CG. The mean age was 67.3 years (SD = 9.3). More than half were female (55.5%), and most of them were married (66.3%). They had an average of two children and a median household net income of 1,501 to 2,000 €. Patients went to school for an average of 9.6 years (SD = 2.0) and most of them completed an apprenticeship. The majority was retired (Table 2).

**Table 2:** Sociodemographic baseline characteristics.

	All	IG			CG	Difference between groups (Sign. (p))		
		ITT-1	ITT-2	AT		ITT-1	ITT-2	AT
Number (n)	4,283	3,061	1,767	1,443	1,222	4,283	2,989	2,665
Age (Mean (SD))	67.28 (9.336)	66.9 (9.260)	66.29 (9.559)	66.60 (9.156)	67.28 (9.523)	.238	.006**	.063
Sex (% female)	55.5%	54.7%	54.4%	55.9%	57.5%	.097	.089	.411
Marital status (n (%))						.743	.127	.052
Married	66.3%	66.2%	66.0%	65.7%	66.5%			
Number of children (Mean (SD))	1.98 (1.053)	2.03 (1.020)	2.06 (1.035)	2.03 (.999)	1.85 (1.116)	< .001**	< .001**	< .001**
Net income^A^ (n (%))						.724	.628	.239
< 500 €	2.9%	2.8%	2.7%	2.1%	3.2%			
500–1000 €	16.9%	16.3%	16.0%	16.1%	18.1%			
1001–1500 €	27.7%	27.0%	27.9%	28.7%	25.2%			
1501–2000 €	22.5%	22.4%	22.5%	22.9%	22.7%			
2001–2500 €	16.3%	16.2%	16.5%	16.7%	16.7%			
2501–3000 €	6.8%	7.0%	7.3%	7.1%	6.4%			
3001–3500 €	3.8%	3.7%	3.6%	3.6%	4.0%			
> 3501 €	3.8%	3.9%	3.6%	2.8%	3.7%			
Years of school (Mean (SD))	9.6 (1.988)	9.64 (1.962)	9.61 (1.926)	9.59 (1.906)	9.69 (2.050)	.427	.279	.190
Education (n (%))						< .001**	< .001**	< .001**
Apprenticeship	63.9%	68.4%	70.6%	70.8%	54.5%			
Technical college	14.1%	11.3%	11.5%	11.2%	19.9%			
University	6.0%	5.0%	4.4%	4.5%	8.2%			
Other	6.9%	6.7%	6.8%	6.5%	7.2%			
None	9.1%	8.5%	6.8%	7.0%	10.2%			
Occupation (n (%))						.451	.862	.495
Occupied	10.5%	10.4%	11.4%	11.2%	10.6%			
Unemployed	3.7%	3.6%	3.8%	3.1%	4.0%			
Homemaker	3.4%	3.3%	3.5%	3.6%	3.5%			
Retired	73.9%	74.5%	72.3%	72.6%	72.5%			
Early retirement	5.3%	5.2%	5.7%	6.4%	5.3%			
Incapacitated for work	3.3%	2.9%	3.3%	3.2%	4.2%			
Campaign						.438	.177	.188
Chronic C. 1	23.2%	23.2%	21.0%	24.9%	22.2%			
Chronic C. 2	63.1%	63.2%	63.6%	58.3%	63.7%			
Heart Failure C.	9.3%	9.3%	10.9%	11.9%	9.4%			
Mental health C.	4.4%	4.3%	4.5%	4.9%	4.7%			

IG = intervention group; CG = control group; ITT-1 = intention-to-treat analysis 1; ITT-2 = intention-to-treat analysis 2; AT = as treated analysis.

^A^ = per month

** = significant difference between the groups p < .01

There were statistically significant differences between the IG and the CG for the primary and the sensitivity analysis (ITT-1, ITT-2, and AT). The IG had significantly more children than the CG although we considered the difference as only statistically but not clinically significant. Nevertheless, there was a difference between the groups regarding their level of education that was both statistically and clinically significant (p < .001). Participants more likely completed an apprenticeship than decliners and patients in the CG more often had a university degree.

In addition to the sociodemographic characteristics, the IG and CG showed further statistically significant baseline differences. For the ITT-1 analysis, there were differences in the physical subscale of the SF-12 (p = .016), and the reported health status (EQ5D-VAS) (p = .028), each with the IG reporting a slightly higher quality of life. Also, there were significant differences in the ITT-1 analysis for medical adherence (p = .006) with the IG being slightly more adherent, the BMI (p = .007), patient activation (p = .007), for health literacy (p = .004) and distress (p = .019). For the ITT-2 analysis, there were just differences regarding the BMI (p< .001) with the IG reporting a higher BMI than the CG. For the AT analysis, there are difference in the baseline values for the reported alcohol consumption (p = .030), with the IG drinking a bit less, medication adherence (p = .041), with the IG being slightly more adherent, and the BMI (p = .001) with the IG reporting a higher BMI (Table 3). All in all it can be subsumed, that the differences might be statistically significant, but the clinical significance is questionable.

**Table 3:** Clinical baseline characteristics.

	All	IG			CG	Difference between groups (Sign. (p))		
		ITT-1	ITT-2	AT		ITT-1	ITT-2	AT
Number (N)	4283	3061	1767	1443	1222	4283	2989	2665
**Quality of life**								
SF-12 Mental Subscale	41.52 (6.22)	41.43 (6.20)	41.43 (6.13)	41.47 (6.12)	41.77 (6.30)	.140	.173	.256
SF-12 Physical Subscale	36.48 (11.27)	36.76 (11.26)	36.02 (10.74)	35.90 (10.63)	35.77 (11.28)	.016*	.557	.772
Health status (EQ5D-VAS)	55.43 (20.41)	55.87 (20.20)	54.82 (19.89)	54.93 (19.59)	54.32 (20.91)	.028*	.516	.449
**Health behaviors**								
Alcohol consumption (AUDIT-C)	2.17 (2.00)	2.14 (1.98)	2.14 (2.01)	2.06 (1.99)	2.25 (2.02)	.130	.192	.030*
Smoking (%)	15.5%	15.3%	15.8%	14.9%	16.2%	.510	.798	.416
Physical activity (hours per week)	8.38 (10.01)	8.54 (10.24)	8.27 (9.87)	8.33 (10.09)	7.98 (9.41)	.103	.421	.367
Physical activity (metabolic rate per week)	4231 (5117)	4301 (5278)	4155 (5033)	4169 (5121)	4055 (4687)	.157	.585	.552
BMI (kg/m^2^)	28.86 (5.48)	29.01 (5.53)	29.28 (5.72)	29.27 (5.71)	28.50 (5.36)	.007**	< .001**	.001**
Medication adherence (MARS-D)	24.09 (1.66)	24.14 (1.62)	24.09 (1.59)	24.11 (1.57)	23.98 (1.75)	.006**	.089	.041*
Measuring blood pressure	3.64 (1.42)	3.62 (1.42)	3.61 (1.41)	3.60 (1.41)	3.70 (1.42)	.141	.101	.081
Measuring blood sugar	1.80 (1.11)	1.81 (1.11)	1.79 (1.11)	1.79 (1.11)	1.78 (1.12)	.397	.752	.723
Foot monitoring self	2.56 (1.13)	2.55 (1.12)	2.54 (1.11)	2.52 (1.11)	2.59 (1.14)	.302	.235	.201
Foot monitoring by physician	1.80 (0.82)	1.79 (0.82)	1.82 (0.82)	1.83 (0.82)	1.80 (0.81)	.173	.571	.387
**Psychosocial Outcomes**								
Patient Activation (PAM)	39.12 (5.32)	39.27 (5.30)	38.91 (5.20)	38.90 (5.15)	38.77 (5.38)	.007**	.481	.531
Health Literacy (FCCHL)	32.67 (6.85)	32.85 (6.83)	32.41 (6.57)	32.45 (6.53)	32.24 (6.88)	.011*	.511	.433
Stages of Change (SOC)	12.80 (5.61)	12.76 (5.60)	12.85 (5.43)	12.68 (5.33)	12.90 (5.64)	.473	.817	.318
Anxiety (HADS-A)	10.16 (1.54)	10.13 (1.55)	10.21 (1.54)	10.23 (1.52)	10.23 (1.52)	.051	.700	.963
Depression (HADS-D)	8.53 (1.72)	8.50 (1.70)	8.48 (1.70)	8.46 (1.70)	8.548 (1.79)	.208	.121	.084
Distress (HADS-T)	18.69 (2.20)	18.64 (2.19)	18.69 (2.20)	18.69 (2.19)	18.81 (2.24)	.019*	.139	.165

IG = intervention group; CG = control group; ITT-1 = intention-to-treat analysis 1; ITT-2 = intention-to-treat analysis 2; AT = as treated analysis; Means (SD) provided unless specified otherwise.

* = significant difference between the groups p < .05

** = significant difference between the groups p < .01

### 3.3 Outcomes

In the main analysis, the ITT-2, a mixed model analysis was used to determine the group effect over all times of measurement (‘time x group’) controlling for level of education and age (Table 4). The estimated marginal means, their standard errors and the contrasts between baseline and t_1_, t_2_ and t_3_ for ITT-2 are presented in Table 5, accompanied by the differences in the contrasts between the groups, showing the differences in differences.

**Table 4:** Mixed model analysis (ITT-1, ITT-2, AT), overall effects (‘time x group’) adjusted for education and age.

	ITT-2^A^ (main analysis)	ITT-1^B^	AT^B^
	Sign. (p)	Sign. (p)	Sign. (p)
**Quality of life**			
SF-12 Mental Subscale	0.963	0.91	0.82
SF-12 Physical Subscale	0.441	0.56	0.41
Health status (EQ5D-VAS)	0.147	0.48	0.29
**Health behaviors**			
Alcohol consumption (AUDIT-C)	0.238	0.47	0.40
Smoking	0.531	0.61	0.87
Physical activity (hours per week)	0.030*	0.01*	0.03*
Physical activity (metabolic rate per week)	0.048*	0.01*	0.04*
Body Mass Index (BMI; kg/m^2^)	0.009**	0.59	0.01**
Adherence (MARS-D)	0.939	0.89	0.98
Measuring blood pressure	<0.001***	<0.01**	< .0001**
Measuring blood sugar	0.619	0.93	0.78
Foot monitoring self	0.352	0.27	0.38
Foot monitoring by physician	0.720	0.60	0.57
**Psychosocial outcomes**			
Patient activation (PAM)	<0.001***	0.56	< 0.01**
Health literacy (FCCHL)	<0.001***	0.08	< .0001**
Stages of Change (SOC)	0.005**	0.02*	< 0.01**
Anxiety (HADS-A)	0.646	0.77	0.55
Depression (HADS-D)	0.758	0.51	0.86
Distress (HADS-T)	0.815	0.73	0.92

* = significant p≤.05

** = significant p≤.01

^**A**^ = adjusted for education and age

^**B**^ = adjusted for education

**Table 5:** Estimated marginal means, their standard errors and contrasts between baseline (t_0_) and t_1_, t_2_, t_3_ for ITT-2 (intervention group and control group); differences in contrasts between the groups.

			Intervention group						Control group						Differences in differences		
					Difference to baseline		Signifi-cance	Effect size			Difference to baseline		Signifi-cance	Effect size	Differences in contrasts between the groups		Signifi-cance
			n	EMM (SE)	Contrasts (adjusted EMM differences)	95%-CI	(p)	Cohen’s d	n	EMM (SE)	Contrasts (adjusted EMM differences)	95%-CI	(p)	Cohen’s d	Contrasts (adjusted group contrasts)	95%-CI	(p)
**Quality of life**	SF-12 Mental Subscale	t_0_	1539	42.63 (0.86)					1033	42.97 (0.86)							
		t_1_	1101	43.08 (0.88)	0.45	(-0.27;1.17)	0.22	0.07	760	42.83 (0.88)	-0.15	(-094;0.65)	0.72	-0.02	0.60	-0.48;1.67	0.27
		t_2_	945	42.95 (0.89)	0.33	(-0.43;1.08)	0.40	0.05	602	42.79 (0.90)	-0.19	(-1.05;0.67)	0.67	-0.03	0.51	-0.63;1.65	0.38
		t_3_	787	42.63 (0.90)	0.01	(-0.80;0.82)	0.99	0.00	514	42.63 (0.91)	-0.35	(-1.26;0.57)	0.46	-0.06	0.35	-0.87;1.58	0.57
	SF-12 Physical Subscale	t_0_	1539	34.08 (0.99)					1033	33.67 (1.00)				-0.02			
		t_1_	1101	34.97 (1.00)	0.89	(0.21;1.46)	< .01**	0.08	760	34.69 (1.00)	1.02	(0.38;1.65)	< .01**	0.09	0.13	-0.98;0.72	0.77
		t_2_	945	35.03 (1.00)	0.94	(0.35;1.54)	< .01**	0.09	602	34.38 (1.01)	0.71	(0.03;1.39)	0.04*	0.06	0.23	-0.67;1.19	0.62
		t_3_	787	34.72 (1.01)	0.63	(-0.01;1.28)	0.05*	0.06	514	33.72 (1.02)	0.05	(-0.67;0.78)	0.89	0.00	0.58	-0.39;1.55	0.24
	Health status (EQ5D-VAS)	t_0_	1714	53.46 (1.45)					1171	53.11 (1.45)							
		t_1_	1191	55.74 (1.48)	2.28	(1.12;3.44)	.0001**	0.11	832	53.47 (1.48)	0.36	(-0.93;1.64)	0.59	0.02	1.92	(0.19;3.66)	0.03*
		t_2_	1042	55.71 (1.49)	2.25	(1.03;3.47)	< .001**	0.11	655	53.80 (1.51)	0.69	(-0.70;2.09)	0.33	0.03	1.56	(0.30;3.41)	0.10
		t_3_	848	54.72 (1.52)	1.26	(-0.06;2.59)	0.06	0.06	565	53.17 (1.53)	0.06	(-1.41;1.54)	0.93	0.00	1.20	(0.78;3.18)	0.24
**Health behaviors**	Alcohol consumption (AUDIT-C)	t_0_	1477	1.93 (0.16)					1046	2.07 (0.16)					-08		
		t_1_	1180	1.69 (0.16)	-0.25	(-0.34;-0.16)	< .0001**	-0.12	824	1.90 (0.16)	-0.17	(-0.26;-0.07)	< .001**	-0.08	-0.08	(-0.21;0.05)	0.23
		t_2_	824	1.81 (0.17)	-0.12	(-0.22;-0.02)	0.02*	-0.06	539	2.00 (0.17)	-0.07	(-0.18;0.04)	0.20	-0.03	-0.05	(-0.20;0.10)	0.52
		t_3_	802	1.58 (0.17)	-0.35	(-0.45;-0.25)	< .0001**	-0.17	544	1.87 (0.17)	-0.20	(-0.31;-0.08)	< .001**	-0.10	-0.15	(-0.31;0.00)	0.05
	Smoking	t_0_	1740	1.82 (0.02)					1201	1.81 (0.02)							
		t_1_	1220	1.84 (0.02)	0.01	(0.00;0.03)	0.03*	0.03	841	1.83 (0.02)	0.02	(0.00;0.03)	0.01**	0.05	-0.01	(.0.02;0.02)	0.68
		t_2_	995	1.85 (0.02)	0.03	(0.01;0.04)	.0001**	0.08	622	1.83 (0.02)	0.02	(0.00;0.04)	0.01**	0.05	0.01	(-0.01;0.03)	0.43
		t_3_	821	1.83 (0.02)	0.01	(-0.01;0.02)	0.42	0.03	547	1.83 (0.02)	0.02	(-0.00;0.03)	0.07	0.05	-0.01	(-0.03;0.01)	0.41
	Physical activity (hours per week)	t_0_	1767	6.87 (0.59)					1222	6.95 (0.59)							
		t_1_	1251	6.61 (0.61)	-0.27	(-0.85;0.31)	0.37	-0.03	858	7.43 (0.61)	0.48	(-0.17;1.12)	0.15	0.05	-0.74	(-1.61;0.12)	0.10
		t_2_	1081	6.81 (0.62)	-0.06	(-0.67;0.55)	0.85	-0.01	676	6.87 (0.63)	-0.08	(-0.78;0.62)	0.81	-0.01	0.03	(-0.90;0.96)	0.95
		t_3_	870	6.94 (0.63)	0.07	(-0.60;0.73)	0.84	0.01	580	6.21 (0.64)	-0.75	(-1.49;-0.01)	<0.05*	-0.08	0.81	(-0.18;1.81)	0.11
	Physical activity (metabolic rate per week)	t_0_	1767	3288 (346)					1222	3323 (346)							
		t_1_	1251	3363 (354)	75.69	(-237;389)	0.64	0.02	858	3801 (356)	478.84	(133;825)	<0.01**	0.10	-403.15	(-876;63)	0.09
		t_2_	1081	3468 (358)	180.64	(-148;509)	0.28	0.04	676	3452 (364)	129.31	(-247;505)	0.50	0.03	51.33	(-448;550)	0.84
		t_3_	870	3360 (365)	72.83	(-285;430)	0.69	0.01	580	3024 (370)	-298.13	(-696;100)	0.14	-0.06	370.96	(-164;906)	0.17
	Body Mass Index (BMI)	t_0_	1647	27.90 (0.43)					1151	27.27 (0.43)							
		t_1_	1154	27.75 (0.43)	-0.15	(-0.31;0.01)	0.07	-0.03	797	27.43 (0.43)	0.15	(-0.09;0.33)	0.10	0.03	-0.30	(-0.55;-0.06)	0.02*
		t_2_	1030	27.74 (0.43)	-0.16	(-0.33;0.01)	0.07	-0.03	654	27.53 (0.43)	0.26	(0.06;0.45)	0.01**	0.05	-0.41	(-0.67;-0.16)	0.002*
		t_3_	827	27.69 (0.43)	-0.21	(-0.40;-0.03)	0.02*	-0.04	554	27.36 (0.43)	0.09	(-0.11;0.30)	0.38	0.02	-0.31	(-0.58;-0.03)	0.03*
	Adherence (MARS-D)	t_0_	1683	24.01 (0.12)					1152	23.88 (0.12)							
		t_1_	1189	24.03 (0.12)	0.02	(-0.08;0.11)	0.74	0.01	824	23.90 (0.12)	0.03	(-0.08;0.13)	0.64	0.02	-0.01	(-0.15;0.16)	0.90
		t_2_	996	24.05 (0.12)	0.04	(-0.06;0.15)	0.40	0.03	630	23.95 (0.12)	0.07	(-0.05;0.19)	0.25	0.04	-0.02	(-0.18;0.13)	0.75
		t_3_	834	24.08 (0.12)	0.07	(-0.04;0.18)	0.19	0.04	547	23.92 (0.12)	0.04	(-0.08;0.17)	0.50	0.02	0.31	(-0.13;0.20)	0.71
	Measuring blood pressure	t_0_	1678	2.63 (0.07)					1146	2.47 (0.07)							
		t_1_	1143	2.81 (0.07)	0.18	(0.11;0.24)	< .0001**	0.15	799	2.46 (0.07)	-0.01	(-0.09;0.06)	0.73	-0.01	0.19	(0.09;029)	< .001**
		t_2_	1006	2.70 (0.07)	0.07	0.00;0.14)	0.04*	0.06	621	2.43 (0.07)	-0.04	(-0.12;0.04)	0.29	-0.04	0.11	(0.01;0.22)	0.03*
		t_3_	815	2.66 (0.07)	0.03	(-0.04;0.11)	0.41	0.03	549	2.50 (0.07)	0.03	(-0.06;0.11)	0.52	0.03	0.00	(-0.10;0.12)	0.93
	Measuring blood sugar	t_0_	1576	1.52 (0.06)					1093	1.51 (0.06)							
		t_1_	1112	1.55 (0.06)	0.03	(-0.01;0.01)	0.09	0.03	781	1.51 (0.06)	0.00	(-0.04;0.04)	0.97	0.00	0.03	(-0.02;0.09)	0.27
		t_2_	981	1.56 (0.06)	0.04	(0.00;0.08)	0.03*	0.04	617	1.52 (0.06)	0.01	(-0.04;0.05)	0.68	0.01	0.03	(-0.02;0.09)	0.25
		t_3_	758	1.58 (0.03)	0.06	(0.02;0.11)	<0.01**	0.05	506	1.56 (0.06)	0.05	(0.00;0.10)	0.05*	0.04	0.02	(-0.05;0.08)	0.61
	Foot monitoring self	t_0_	1684	2.48 (0.08)					1150	2.45 (0.08)							
		t_1_	1162	2.51 (0.08)	0.04	(-03;0.10)	0.32	0.03	807	2.43 (0.08)	-0.03	(-0.10;0.05)	0.52	-0.03	0.06	(-0.04;0.16)	0.25
		t_2_	1027	2.53 (0.08)	0.05	(-0.02;0.12)	0.20	0.04	635	2.43 (0.08)	-0.02	(-0.11;0.06)	0.56	-0.02	0.07	(-0.04;0.18)	0.20
		t_3_	821	2.60 (0.08)	0.12	(0.04;0.20)	< .01**	0.10	545	2.48 (0.08)	0.02	(-0.06;0.11)	0.59	0.02	0.10	(-0.02;0.21)	0.10
	Foot monitoring by physician	t_0_	1499	1.66 (0.04)					1028	1.64 (0.04)							
		t_1_	1076	1.66 (0.05)	0.00	(-0.05;0.05)	0.97	0.00	721	1.60 (0.05)	-0.04	(-0.09;0.02)	0.22	-0.05	0.04	(-0.04;0.11)	0.38
		t_2_	920	1.68 (0.05)	0.02	(-0.03;0.07)	0.46	0.02	550	1.63 (0.05)	-0.01	(-0.07;0.06)	0.84	-0.01	0.03	(-0.06;0.11)	0.53
		t_3_	749	1.69 (0.5)	0.03	(-0.03;0.09)	0.29	0.04	471	1.62 (0.05)	-0.01	(-0.08;0.05)	0.66	-0.01	0.05	(-0.04;0.13)	0.30
**Psychosocial outcomes**	Patient activation (PAM)	t_0_	1671	38.40 (0.31)					1156	38.47 (0.30)							
		t_1_	1189	39.11 (0.31)	0.71	(0.39;1.03)	< .0001**	0.14	818	38.23 (0.32)	-0.24	(-0.60;0.11)	0.18	-0.04	0.95	(0.48;1.43)	< .0001**
		t_2_	1038	38.99 (0.32)	0.59	(0.25;0.93)	< .001**	0.11	642	38.37 (0.33)	-0.10	(-0.49;0.29)	0.61	-0.02	0.69	(0.18;1.20)	0.01*
		t_3_	843	38.57 (0.33)	0.17	(-0.20;0.53)	0.37	0.03	559	38.27 (0.33)	-0.20	(-0.60;0.21)	0.34	-0.04	0.37	(0.18;0.91)	0.19
	Health literacy (FCCHL)	t_0_	1684	32.59 (0.35)					1156	32.71 (0.35)							
		t_1_	1187	33.45 (0.36)	0.86	(0.47;1.24)	< .0001**	0.13	812	32.54 (0.36)	-0.16	(-0.59;0.27)	0.46	-0.02	1.02	(0.44;1.60)	< .001**
		t_2_	1009	33.51 (0.37)	0.92	(0.51;1.33)	< .0001**	0.14	639	32.03 (0.38)	-0.67	(-1.14;-0.21)	< .01**	-0.10	1.60	(0.97;2.22)	< .0001**
		t_3_	812	33.48 (0.38)	0.89	(0.44;1.33)	.0001**	0.14	554	32.07 (0.38)	-0.63	(-1.13;-0.14)	0.01**	-0.09	1.52	(0.86;2.19)	< .001**
	Stages of Change (SOC)	t_0_	1723	13.33 (0.32)					1192	13.52 (0.31)							
		t_1_	1220	12.75 (0.32)	-0.58	(-0.87;-0.29)	.0001**	-0.11	847	13.58 (0.32)	0.06	(-0.26;0.37)	0.72	0.01	-0.64	(-1.07;-0.21)	< .01**
		t_2_	1057	12.70 (0.33)	-0.63	(-0.93;-0.33)	< .0001**	-0.12	661	13.52 (0.33)	0.00	(-0.34;0.35)	1.00	0.00	-0.63	(-1.08;-0.17)	< .01**
		t_3_	860	12.67 (0.33)	-0.66	(-098;-0.33)	.0001**	-0.12	565	13.56 (0.34)	0.04	(-0.32;0.41)	0.82	0.01	-0.70	(-1.19;-0.21)	< .01**
	Anxiety (HADS-A)	t_0_	1744	10.32 (0.14)					1205	10.30 (0.14)							
		t_1_	1243	10.30 (0.15)	-0.02	(-0.12;0.09)	0.78	-0.01	848	10.37 (0.15)	0.07	(-0.04;0.19)	0.22	0.05	-0.09	(-0.25;0.07)	0.27
		t_2_	1072	10.35 (0.15)	0.03	(-0.08;0.14)	0.54	0.02	669	10.37 (0.15)	0.07	(-0.06;0.19)	0.30	0.05	-0.03	(-0.20;0.14)	0.71
		t_3_	862	10.48 (0.15)	0.16	(0.04;0.28)	<0.01**	0.10	576	10.44 (0.15)	0.14	(0.01;0.28)	0.03*	0.09	0.02	(-0.16;0.20)	0.85
	Depression (HADS-D)	t_0_	1744	8.43 (0.09)					1205	8.56 (0.09)							
		t_1_	1243	8.57 (0.09)	0.15	(0.03;0.26)	0.01**	0.09	848	8.63 (0.09)	0.07	(-0.05;0.20)	0.26	0.04	0.07	(-0.10;0.24)	0.40
		t_2_	1072	8.51 (0.09)	0.09	(-0.03;0.21)	0.16	0.05	669	8.60 (0.09)	0.04	(-0.09;0.18)	0.54	0.02	0.04	(-0.14;0.22)	0.64
		t_3_	862	8.49 (0.09)	0.06	(-0.07;0.19)	0.33	0.04	576	8.53 (0.10)	-0.03	(-0.17;0.11)	0.68	-0.02	0.09	(-0.10;0.29)	0.34
	Distress (HADS-T)	t_0_	1744	18.74 (0.17)					1205	18.86 (0.17)							
		t_1_	1243	18.87 (0.17)	0.13	(-0.02;0.22)	0.09	0.06	848	19.00 (0.17)	0.14	(-0.02;0.21)	0.09	0.06	-0.01	(-0.24;0.21)	0.90
		t_2_	1072	18.86 (0.17)	0.12	(-0.04;0.28)	0.14	0.05	669	18.97 (0.18)	0.11	(-0.07;0.29)	0.24	0.05	0.01	(-0.23;0.25)	0.94
		t_3_	862	18.97 (0.18)	0.23	(0.05;0.40)	0.01**	0.10	576	18.97 (0.18)	0.12	(-0.08;0.31)	0.24	0.05	0.11	(-0.15;0.37)	0.40

EMM = Estimated Marginal Means.

* = significant difference p < .05

** = significant difference p < .01

#### 3.3.1 Quality of life

Overall, groups did not statistically significant differ regarding the course of **mental** (p = .963) and **physical quality of life** (p = .441) from baseline to three years (SF-12; interaction effect ‘time x group’). Also, there was no significant difference between the groups over time concerning the **health status** reported with the visual analogue scale of the EQ5D (p = .147).

#### 3.3.2 Health behaviors

For health behaviors, there was no difference between the groups over time (‘time x group’) regarding **alcohol consumption** (p = .238), **smoking** (p = .531), **medication adherence** (p = .939), **measuring blood sugar** (p = .619), **foot monitoring by themselves** (p = .352), and **foot monitoring by a physician** (p = .720). Nevertheless, there was a significant ‘time x group’ interaction effect regarding the **physical activity** in *hours per week* (p = .030), although the ‘time x group’ interaction contrasts (adjusted group contrasts) between the groups were all non-significant. There was also a significant ‘time x group’ interaction effect regarding **physical activity** measured *in metabolic rate per week* (p = .048), which did not result in any significant difference in contrasts between the groups at any follow-up. Furthermore, there was a significant ‘time x group’ interaction effect on the **BMI** (p = .009). The ‘time x group’ interaction contrasts (adjusted group contrasts) were significant at t_1_ between IG (adjusted EMM Diff = -0.15) and CG (adjusted EMM Diff = 0.15) by -0.30 BMI points (p = .02), at t_2_ between IG (adjusted EMM Diff = -0.16) and CG (adjusted EMM Diff = 0.26) by -0.41 BMI points (p = .002), and at t_3_ between IG (adjusted EMM Diff = -0.21) and CG (adjusted EMM Diff = 0.09) by -0.31 BMI points (p = .03). Also, there was a significant ‘time x group’ interaction effect on **measuring blood pressure** (p < .001). The between-group differences in differences (‘time x group’ interaction contrasts) were statistically significant (p < .001) in the changes from baseline at t_1_ between IG (adjusted EMM Diff = 0.18) and CG (adjusted EMM Diff = -0.01) with an adjusted group contrast of .19 and at t_2_ (p = .03) with an adjusted group contrast of .11 (IG: adjusted EMM Diff = 0.07; CG: adjusted EMM Diff = -0.04).

#### 3.3.3 Psychosocial outcomes

For psychosocial outcomes, there were several significant ‘time x group’ interaction effects: For instance, there is an overall effect on **patient activation** (p< .001), resulting in significant ‘time x group’ interaction contrasts in the changes from baseline to t_1_ (p < .0001) with an adjusted group contrast of 0.95 (IG: adjusted EMM Diff = 0.71; CG: adjusted EMM Diff = -0.24), and also in the changes from baseline to t_2_ (p = .01) with an adjusted group contrast of .69 (IG: adjusted EMM Diff = 0.59; CG: adjusted EMM Diff = -0.10). Also, there was a significant overall ‘time x group’ effect for **health literacy** (p < .001), with significant ‘time x group’ interaction contrasts (adjusted group contrasts) in the changes from baseline to t_1_ of 1.02 (p < .001; IG: adjusted EMM Diff = 0.86; CG: adjusted EMM Diff = -0.16)), to t_2_ of 1.60 (p < .0001, IG: adjusted EMM Diff = 0.92; CG: adjusted EMM Diff = -0.67)) and to t_3_ of 1.52 (p < .001; IG: adjusted EMM Diff = 0.89; CG: adjusted EMM Diff = -0.63)). Regarding **stages of change** there is a significant ‘time x group’ effect (p = .005). The ‘time x group’ interaction contrasts were significant for the change from t_0_ to t_1_ with an adjusted group contrast of -0.64 (p < .01) between IG (adjusted EMM Diff = -0.58) and CG (adjusted EMM Diff = 0.06), from t_0_ to t_2_ with an adjusted group contrast of -0.63 (p < .0; IG: adjusted EMM Diff = -0.63; CG: adjusted EMM Diff = 0.00) and from t_0_ to t_3_ with an adjusted group contrast of -0.70 (p < .01; IG: adjusted EMM Diff = -0.66; CG: adjusted EMM Diff = 0.04). Regarding **anxiety** (p = .646), **depression** (p = .758) and **mental distress** (p = .815) there were no significant differences between the groups over the time of three years.

A closer look at the changes from baseline (contrasts (adjusted EMM differences)) for both groups may give a more detailed impression of the different courses over time for each outcome (Table 5). There are statistically significant within-group changes from baseline in both groups. Nevertheless, the effect size of all contrasts were below .2 indicating a small effect size.

The detailed results for ITT-2 including the estimated marginal means (EMM), the standard error (SE), the adjusted EMM differences between t_0_ and each follow-up (including 95% confidence interval (CI)), the significance (p) of the adjusted EMM difference, as well as the effect size (Cohen’s d) for each time of measurement for each group, as well as the between-group differences in the within-group changes (adjusted group contrasts), its 95% CI and significance (p) can be found in Table 5. The observed means and standard deviations for all analyses (ITT-1, ITT-2, AT) across all time points can be found in S3 Table.

Estimated marginal means, their standard errors and estimated marginal differences by time (t_0_, t_1_, t_2_, t_3_), adjusted for education for ITT-1 and AT can be found in S4 and S5 Tables.

The **ITT-1** analysis comparing the IG including those declining the TBHC to the CG controlling for level of education showed statistically significant differences in physical activity in hours per week (F_3, 6431.5_ = 3.66; p = .01), physical activity measured in metabolic rate per week (F_3, 6493.0_ = 3.77; p = .01), measuring blood pressure (F_3, 5728.3_ = 3.89; p< .01) and stages of change (F_3, 5955.5_ = 3.37; p = .02) (Table 4).

Additionally, we employed an **as-treated** approach (AT) comparing those participants that received five or more calls to the CG (Table 4). Controlling for level of education, there were statistically significant ‘time x group’ interaction effects in physical activity in hours per week (F_3, 4427.0_ = 3.11; p = .03), physical activity measured in metabolic rate per week (F_3, 4459.1_ = 2.76; p = .04), body mass index (F_3, 3827.2_ = 4.12; p = .01), measuring blood pressure (F_3, 3997.1_ = 7.65 p< .0001). Following psychosocial outcomes showed significant interaction effects: Patient activation (F_3, 4121.0_ = 5.11; p< .01), health literacy (F_3, 4038.2_ = 9.69; p< .0001), and stages of change (F_3, 4169.9_ = 5.08; p< .01).

#### 3.3.4 Moderator analyses

We conducted moderator analyses to examine whether intervention effects vary among campaigns (“heart failure campaign” (N = 397), “chronic campaign 1” (N = 993), “chronic campaign 2” (N = 2698), and “mental health campaign” (N = 190)). We found campaign-specific intervention effects for foot monitoring by physician (p = .036) and blood sugar measurements (p = .001). Detailed results of the subgroup analyses can be found in Table 6. The estimated marginal means for “measuring blood sugar” and “foot monitoring by physician” can be found in S6 Table.

**Table 6:** Mixed model analysis (ITT-2), overall effects (‘time x group x campaign’) adjusted for education and age.

	Df	F	Sign. (p)
**Quality of life**			
SF-12 Mental Subscale	12, 4414.8	1.00	0.443
SF-12 Physical Subscale	12, 3919.7	0.62	0.827
Health status (EQ5D-VAS)	12, 4440.9	0.67	0.782
**Health behaviors**			
Alcohol consumption (AUDIT-C)	12, 3755.1	0.97	0.476
Smoking	12, 4111.5	1.49	0.118
Physical activity (hours per week)	12, 4735.9	1.16	0.303
Physical activity (metabolic rate per week)	12, 4765.0	1.03	0.415
Body Mass Index (BMI; kg/m^2^)	12, 4046.3	1.07	0.380
Adherence (MARS-D)	12, 4327.8	1.72	0.057
Measuring blood pressure	12, 4259.8	0.97	0.476
Measuring blood sugar	12, 3821.8	2.67	0.001**
Foot monitoring self	12, 4381.9	1.69	0.063
Foot monitoring by physician	12, 3849.3	1.85	0.036*
**Psychosocial outcomes**			
Patient activation (PAM)	12, 4386.8	1.11	0.351
Health literacy (FCCHL)	12, 4308.6	1.26	0.234
Stages of Change (SOC)	12, 4445.8	0.89	0.562
Anxiety (HADS-A)	12, 4776.8	0.74	0.715
Depression (HADS-D)	12, 4704.0	0.87	0.574
Distress (HADS-T)	12, 4769.8	0.81	0.646

Df = degrees of freedom; F = F Value.

* = significant p < .05

** = significant p < .01

## 4. Discussion

### 4.1 Principal findings

The aim of this randomized controlled trial was to evaluate the effectiveness of a TBHC intervention for people living with chronic conditions on a variety of patient reported outcomes such as QoL, health behaviors, and psychosocial outcomes. We compared the effectiveness of a TBHC intervention for people living with chronic conditions, insured at a German statutory health insurance, to a usual care group.

In the a modified intention-to-treat analysis, comparing TBHC participants and CG (ITT-2), seven out of 19 outcomes showed significant overall intervention effects (‘time x group’) after controlling for education and age. Patients in the IG group differed significantly from patients in the CG group regarding their physical activity (hours per week and metabolic rate per week) and their BMI, which can be attributed to the central focus of the TBHC to promote exercise, to improve nutrition, and to provide information about these topics (see S2 Table ‘TIDIER Checklist’). With regard to blood pressure measurement, TBHC was found to result in more frequent blood pressure measurements, although the overall frequency of measuring blood pressure is significantly lower in the CG at all times. One explanation for this finding is that this is one of the main foci of the TBHC, including the use of a “blood pressure log book” for recording blood pressure measurements. Furthermore, it was found that TBHC results in greater (patient) activation, like taking charge over their health, and better health literacy. This suggests that the MI technique used by the health coaches is an effective counselling method that enhances motivation and thus acts as a catalyst to accelerate health behavior change. Nonetheless, in contrast to these findings, it was found that patients in the TBHC group were less motivated to change according to the stages of change (no willingness to change, considering change, preparation, taking action and maintenance) over time compared to patients in the CG whose willingness did not change. One explanation for this finding might be that participants receiving TBHC are more satisfied with their own health behavior and therefore see no reason to change. These findings are supported by a closer look at the contrasts comparing each follow-up to the baseline. The IG remains rather stable regarding their physical activity, whereas the CG shows a significant decrease in active hours per week. Nevertheless, comparing the groups regarding their differences from baseline there is no significant difference between the groups. With regards to the BMI the IG shows a continuous drop over the years, while the CG shows an increase in BMI (especially at the 2 years follow-up). This difference from baseline is also statistically different between the groups at all times. Concerning the overall frequency of measuring blood pressure and the patient activation the IG reveals a short- (t_1_) and mid-term (t_2_) increase that does not remain until t_3_. The change from baseline is also statistically significant between the groups for t_1_ and t_2_. The significant improvement in health literacy in the IG remains stable over all follow-ups, whereas the CG shows a significant decline of health literacy at t_2_ and t_3_, which is also statistically significant in the post hoc interaction contrasts. The decrease of the stages of change in the IG remains stable over all follow-ups, while there is no statistically significant change in the CG. The difference in difference analysis also supports this finding by showing statistically significant group differences in change over all times. These inconsistent findings warrant further investigation in future clinical trials.

Finally, the absence of significant effects requires some comment. There were no effects on QoL (mental, physical QoL and health status), alcohol consumption, smoking, medication adherence, the amount of measuring blood sugar and foot monitoring (by the participants or a physician), anxiety, depression and mental distress. One plausible explanation for the absence of significant effects could be that a change in QoL, health status, anxiety and depression depends on too many other complex and multifaceted factors to be merely influenced by TBHC and the engagement in the desired health behaviors alone such as regular exercise and a healthy diet. The change of addictive behaviors like smoking or alcohol consumption might be too challenging for a broad telephone intervention that focuses on more than one health behavior. The absent effect on medication adherence might be due to problem of the high ceiling effects of the MARS-D and possible effects of social desirability as the TBHC was conducted by their health insurance.

There were just two moderator effects between the study group and the campaigns on ‘blood sugar measurement’ and ‘foot monitoring by a physician’, suggesting limited variation in the treatment effects across campaigns.

The results of the AT analysis were very similar with slightly higher effect sizes. Therefore, we cannot state a dose-effect based on these analyses. There is future research needed to provide valid information on this.

### 4.2 Results in relation to similar studies

Our findings are in line with previous results that report no effect of TBHC on QoL [17, 21, 28, 67, 68], health status [26, 28] or health behaviors such as alcohol consumption [28, 39, 40] and smoking [17, 25, 27, 28, 32, 41]. Likewise, the lack of effects on depression and distress is comparable with the findings of similar studies [17, 19, 25, 27, 28]. Regarding patient activation, findings are consistent with previous studies showing a positive effect of TBHC on patient activation [21, 69].

In contrast with earlier findings in the literature, our findings do not confirm previous research reporting TBHC effects on anxiety [25, 26], which can be attributed to a different focus of our TBHC intervention (S2 Table). However, we found an effect of TBHC on the frequency of blood pressure measurements, physical activity and the BMI of participants, while most studies do not show effects on these parameters (blood pressure measurement [29, 34, 35, 38], physical activity [23, 29–37], BMI [29–31, 38–40, 70–72]), although there are also studies that have similar results (blood pressure measurement [35, 38, 70], physical activity [25, 28, 30, 37, 38, 41, 70, 72, 73], BMI [17, 25, 74]). However, as our findings are exploratory, we can only assume why we see these effects. One possible reason could be that our intervention is more tailored to the needs of the patients, be it their condition, their leisure time activities or their delegation of responsibility regarding their condition. As we also found that the activation and health literacy is higher in the IG than in the CG, especially in the first year, it is possible that empowering the patient to take responsibility for their health actions is a good way to moderate effects on exercise, diet and BMI.

Furthermore, we tested outcomes that were not used so far. Health literacy has not been assessed in the evaluation of a TBHC so far, therefore our findings may be the first to indicate that TBHC could be an effective measure to promote participants’ health literacy. For the Transtheoretical Model of Change and its stages of change there have only been longitudinal, non-randomized studies that concluded, contrary to our findings no effects of TBHC [27, 32, 75].

Overall, these inconsistent findings indicate that the effectiveness of TBHC remains inconclusive given the spectrum of heterogeneous studies, although the effects of the TBHC intervention on psychosocial outcomes are partly in line with the known literature. That said, the data presented in this paper significantly adds knowledge to the existing body of literature regarding the effectiveness of TBHC. Despite some international and nationwide studies like Birmingham OwnHealth [76, 77], TERVA [78], the DIAL study [24] and the Connection Program [79], there have not been many studies with such a large sample available for the assessment of patient reported outcomes.

### 4.3 Strengths and weaknesses

As this study included 4,283 patients in the analyses, a main strength is the large sample size. There are few studies in this field that include as many patients in the evaluation of patient reported data. The drop-out over this amount of time of 50.6% is as expected as it is comparable to the pilot study [45]. We tried handle missing values conservatively: EM-imputation for item-level missings to compute scores for those that provide 70% of the scores data on the one hand, and the statistical analyses with mixed model analysis which is quite robust against missing values on the other hand.

The randomization process based on Zelen’s single-consent design before informed consent ensures that patients participating in the intervention are clearly willing to take part in the intervention. Also, the participation rate is higher than in classic RCT designs leading to larger sample sizes [80]. Nevertheless, an intention-to-treat analysis, in this case ITT-1, is necessary to avoid selection bias in Zelen’s design. Therefore, the thorough statistical evaluation can be considered a strength of this study.

For good scientific practice a study protocol was published [15], and it was registered in the German Clinical Trials Register (GCTR). The inclusion of more than one of the most important chronic conditions, like diabetes mellitus type II and chronic heart diseases, increases generalizability. Also, this study provides high treatment fidelity, as it is manual-based; the coaches received regular supervision and the quality of the intervention was assessed regularly. A further strength is that this study was conducted in real routine health care. Therefore, generalizability and external validity are high. Also, the analysis of three yearly follow-ups enables the readers to assess the long-term effectiveness of this intervention.

Nevertheless, this study has some limitations. Although we employed an intention-to-treat approach (ITT-1) to avoid the participation bias, one could argue that this is not a flawless ITT, as we do not have complete data of all patients that were randomized, but of those, that replied at least at baseline [81]. Therefore, the results must be interpreted with caution. It is possible that there are differences between those taking part and those who dropped out on the one hand and between those taking part in the intervention and those who declined the intervention on the other hand. As stated earlier, eligible insurants decided whether to participate after randomization—this could be a crucial cause for potential bias. 57.4% of the randomized potential participants decided to decline participation. A comparison between those groups showed that participants were significantly less educated than decliners or CG. Therefore, we tried to control for this bias by adjusting the model by education and age as a fixed effects. Nevertheless, there is a possibility of other differences between the groups, as we did not assess all possible confounding variables. Additionally, we did not assess whether the patients have an immigrant background, which could also lead to some participation and group differences. Also, we lacked the insurants’ clinical and physician data. Therefore, the severity of the patients’ condition could not be considered. In addition, no data were available on the routine health care that has taken place alongside the intervention and during follow-up. Therefore, it was not possible to ascertain how much of a poorly controlled disease status is lack of adequate treatment. Another limitation could be that the chronic campaign was heterogeneous with different diagnoses, like diabetes mellitus type II, coronary artery disease etc. in need for different coaching targets. To prevent an even higher bias within the chronic campaign we divided it into two smaller subgroups that differed regarding their inclusion criteria. Therefore, the campaigns are slightly different to the manuscript reporting the health economic outcome [44].

Also, the questionnaires were sent out by the health insurance. Therefore, it is possible that there is a bias due to social desirability, despite making clear the pseudonomyzation. Also, patient reported outcomes are prone to social desirability bias and inaccurate reporting. Additionally, effect sizes of all statistically significant EMM differences (despite for health literacy and measuring blood pressure) were very small. Also, the clinical significance needs to be questioned as all statistically significant effects were practically very small and we did not adjust for multiple testing (Bonferroni).

Generalizability of the findings might also be limited, since those insured at this health insurance have a slightly higher socioeconomic status than at other health insurances due to historic reasons. Nevertheless, in Germany nearly all citizens are insured for health care as mandatory members of the public health insurance (86.2%) or private health insurances (10.6%) [82].

## 5.Conclusion and practice implications

Based on previous research and the results of our study, TBHC interventions might have small effects on some patient reported outcomes. It would be interesting to find out, which intervention components actually have an effect. So maybe a more disease specific approach could make it easier to distinguish between effective and ineffective components without disease specific variables diluting the results. Also, future research should focus on who exactly profits most from TBHC interventions and whether there are any differences regarding disease, multimorbidity or gender.

## Supporting information

S1 Table CONSORT checklist. (PDF)

S2 Table TIDieR checklist. (PDF)

S3 Table Observed means and standard deviations for all outcomes and measurement times. (PDF)

S4 Table Model-predicted (ITT-1) estimated marginal means, their standard errors and estimated marginal differences by time (t_0_, t_1_, t_2_, t_3_), adjusted for education. (PDF)

S5 Table Model-predicted (AT) estimated marginal means, their standard errors and estimated marginal differences by time (t_0_, t_1_, t_2_, t_3_), adjusted for education. (PDF)

S6 Table Model-predicted (time x group x campaign; ITT-2) estimated marginal means, their standard errors and estimated marginal differences by time (t_0_, t_1_, t_2_, t_3_), adjusted for education and age for “measuring blood sugar” and “foot monitoring by physician”. (PDF)

S1 File Published study protocol. (PDF)

S2 File Study protocol for ethics committee. (PDF)

S3 File Ethics approval document. (PDF)

S1 Fig Analysis principles. (TIFF)
