# Supplementary material for: Effects of telephone-based health coaching on patient-reported outcomes and health behavior change: A randomized controlled trial
Source: PLoS One. 2020 Sep 22;15(9):e0236861. doi: 10.1371/journal.pone.0236861 (PMC7508388; doi:10.1371/journal.pone.0236861)
Supplement: S3 Table — (PDF) [file pone.0236861.s003.pdf]

Supporting information 4. Observed means and standard deviations for all outcomes and measurement times.

			Intervention ITT 1		Intervention ITT2		Intervention AT		Control	
			n	M (SD)	n	M (SD)	n	M (SD)	n	M (SD)
Quality of life	SF-12 Mental Subscale	t_0	2625	41.43 (6.20)	1539	41.43 (6.13)	1253	41.47 (6.11)	1033	41.77 (6.30)
		t_1	1728	41.22 (6.12)	1101	41.33 (6.06)	971	41.47 (6.01)	760	41.50 (6.12)
		t_2	1538	40.95 (5.94)	945	41.11 (5.94)	839	41.14 (5.96)	602	41.37 (6.25)
		t_3	1280	41.31 (6.32)	787	41.66 (6.41)	695	41.61 (6.42)	514	41.72 (6.30)
	SF-12 Physical Subscale	t_0	2625	36.76 (11.26)	1539	36.02 (10.74)	1253	35.90 (10.63)	1033	35.77 (11.28)
		t_1	1728	37.85 (11.31)	1101	37.20 (10.98)	971	36.94 (10.86)	760	37.31 (11.22)
		t_2	1538	38.08 (11.27)	945	37.45 (11.12)	839	37.09 (11.06)	602	37.02 (11.22)
		t_3	1280	37.70 (11.37)	787	36.97 (11.06)	695	36.77 (10.98)	514	36.49 (11.37)
	Health status (EQ5D-VAS)	t_0	2951	55.87 (20.20)	1714	54.82 (19.89)	1405	54.93 (19.59)	1171	54.32 (20.91)
		t_1	1874	57.90 (20.11)	1191	57.52 (20.00)	1048	57.22 (20.12)	832	55.55 (20.64)
		t_2	1682	58.54 (20.28)	1042	58.00 (20.33)	919	57.59 (20.41)	655	56.70 (50.46)
		t_3	1398	57.91 (20.57)	848	57.35 (20.62)	749	57.01 (20.69)	565	56.14 (21.37)
Health behaviors I	Alcohol consumption (AUDIT-C)	t_0	2575	2.14 (2.00)	1477	2.14 (2.01)	1215	2.06 (1.99)	1046	2.25 (2.02)
		t_1	1789	1.84 (1.94)	1180	1.74 (1.91)	1037	1.70 (1.88)	824	1.99 (2.09)
		t_2	1330	2.16 (1.96)	824	2.12 (1.96)	719	2.10 (1.99)	539	2.40 (1.97)
		t_3	1314	1.73 (1.88)	802	1.67 (1.83)	708	1.65 (1.85)	544	1.99 (2.06)
	Smoking	t_0	3006	15.3%	1740	15.8%	1201	16.2%	1201	16.2%
		t_1	1918	12.6%	1220	12.0%	841	11.5%	841	11.5%
		t_2	1616	9.1%	995	8.7%	622	10.6%	622	10.6%
		t_3	1346	11.0%	821	11.2%	547	10.4%	547	10.4%
	Physical activity (hours per week)	t_0	3061	8.54 (10.24)	1767	8.27 (9.87)	1443	8.33 (10.09)	1222	7.98 (9.41)
		t_1	1962	8.30 (9.93)	1251	8.15 (9.62)	1097	8.17 (9.48)	858	8.87 (10.43)
		t_2	1746	8.49 (9.85)	1081	8.37 (9.69)	955	8.26 (9.76)	676	8.39 (10.47)
		t_3	1434	8.80 (10.51)	870	8.67 (10.50)	769	8.66 (10.56)	580	7.83 (9.13)
	Physical activity (metabolic rate per week)	t_0	3061	4301.01 (5278.59)	1767	4155.27 (5033.09)	1443	4169.82 (5121.60)	1222	4055.87 (4687.63)
		t_1	1962	4334.19 (5354.49)	1251	4244.85 (5107.60)	1097	4256.89 (5062.43)	858	4713.13 (5514.40)
		t_2	1746	4502.56 (5540.28)	1081	4419.75 (5300.73)	955	4346.15 (5350.45)	676	4384.18 (5015.81)
		t_3	1434	4414.99 (5452.34)	870	4431.86 (5564.58)	769	4440.28 (5535.12)	580	3975.02 (4451.21)
	Body Mass Index (BMI)	t_0	2850	29.01 (5.53)	1647	29.28 (5.72)	1341	28.27 (5.71)	1151	28.50 (5.36)
		t_1	1811	28.93 (5.57)	1154	29.05 (5.77)	1015	29.09 (5.81)	797	28.60 (5.48)
		t_2	1658	29.01 (6.52)	1030	29.05 (5.61)	905	29.07 (5.62)	654	28.63 (5.59)
		t_3	1357	28.90 (5.61)	827	28.99 (5.37)	728	29.05 (5.38)	554	28.50 (5.39)
	Adherence (MARS-D)	t_0	3893	24.14 (1.62)	1683	24.09 (1.59)	1381	24.11 (1.57)	1152	23.98 (1.75)
		t_1	1861	24.17 (1.55)	1189	24.14 (1.50)	1045	24.17 (1.45)	824	24.00 (1.71)
		t_2	1612	24.21 (1.45)	996	24.20 (1.36)	877	24.24 (1.30)	630	24.05 (1.66)
		t_3	1373	24.26 (1.33)	834	24.22 (1.35)	737	24.26 (1.34)	547	24.07 (1.70)

			Intervention ITT 1		Intervention ITT2		Intervention AT		Control	
			n	M (SD)	n	M (SD)	n	M (SD)	n	M (SD)
**Health behaviors II**	Alcohol consumption (AUDIT-C)	t_0	2882	8.82 (1.18)	1678	2.87 (1.20)	1369	2.88 (1.19)	1146	2.74 (1.11)
		t_1	1795	2.96 (1.15)	1143	3.08 (1.16)	1008	3.14 (1.15)	799	2.75 (1.15)
		t_2	1601	2.88 (1.15)	1006	1.98 (1.15)	886	3.02 (1.14)	621	2.76 (1.12)
		t_3	1354	2.83 (1.13)	815	2.94 (1.14)	718	2.99 (1.13)	549	2.82 (1.09)
	Measuring blood sugar	t_0	2697	1.81 (1.11)	1576	1.79 (1.11)	1285	1.79 (1.11)	1093	1.78 (1.12)
		t_1	1752	1.80 (1.09)	1112	1.80 (1.10)	984	1.80 (1.11)	781	1.75 (1.08)
		t_2	1563	1.80 (1.10)	981	1.81 (1.12)	868	1.81 (1.13)	617	1.75 (1.08)
		t_3	1281	1.81 (1.10)	758	1.85 (1.17)	668	1.85 (1.13)	506	1.80 (1.11)
	Foot monitoring self	t_0	2876	2.59 (1.14)	1684	2.61 (1.14)	1375	2.62 (1.14)	1150	2.57 (1.13)
		t_1	1819	2.63 (1.15)	1162	2.65 (1.15)	1024	2.64 (1.15)	807	2.54 (1.16)
		t_2	1640	2.62 (1.15)	1027	1.66 (1.14)	907	2.66 (1.14)	635	2.53 (1.15)
		t_3	1348	2.71 (1.10)	821	2.74 (1.10)	726	2.76 (1.09)	545	2.59 (1.14)
	Foot monitoring by physician	t_0	2625	1.79 (0.82)	1499	1.82 (0.82)	1225	1.83 (0.82)	1028	1.80 (0.81)
		t_1	1686	1.78 (0.81)	1076	1.81 (0.81)	947	1.82 (0.80)	721	1.77 (0.82)
		t_2	1479	1.80 (0.80)	920	1.84 (0.81)	810	1.85 (0.80)	550	1.80 (0.79)
		t_3	1230	1.80 (0.78)	749	1.85 (0.78)	662	1.88 (0.78)	471	1.79 (0.80)
**Psychosocial outcomes I**	Patient activation (PAM)	t_0	2879	39.27 (5.30)	1671	38.91 (5.20)	1363	38.90 (5.15)	1156	38.77 (5.38)
		t_1	1868	39.36 (5.60)	1189	39.53 (5.63)	1041	39.54 (5.41)	818	38.66 (5.41)
		t_2	1677	39.45 (5.49)	1038	39.43 (5.43)	918	39.39 (5.48)	642	38.94 (5.59)
		t_3	1377	39.19 (5.46)	843	39.19 (5.41)	747	39.13 (5.36)	559	38.87 (5.55)
	Health literacy (FCCHL)	t_0	2873	32.85 (6.83)	1684	32.41 (6.57)	1372	32.45 (6.53)	1156	32.24 (6.88)
		t_1	1857	33.09 (6.50)	1187	33.25 (5.90)	1045	33.23 (5.85)	812	32.29 (6.92)
		t_2	1626	33.04 (6.40)	1009	33.14 (5.87)	895	33.39 (5.88)	639	32.08 (7.35)
		t_3	1337	32.93 (6.32)	812	33.19 (5.87)	716	33.20 (5.72)	554	31.99 (7.62)
	Stages of Change (SOC)	t_0	2974	12.78 (5.60)	1723	12.85 (5.43)	1411	12.68 (5.33)	1192	12.90 (5.64)
		t_1	1907	12.23 (5.26)	1220	12.13 (5.13)	1070	11.96 (5.10)	847	12.84 (5.72)
		t_2	1700	12.13 (5.35)	1057	12.04 (5.10)	931	11.83 (5.02)	661	12.79 (5.52)
		t_3	1404	11.92 (5.04)	860	11.82 (4.80)	759	11.67 (4.72)	565	12.76 (5.92)
	Anxiety (HADS-A)	t_0	3019	10.13 (1.55)	1744	10.21 (1.54)	1422	10.23 (1.52)	1205	10.23 (1.52)
		t_1	1946	10.17 (1.51)	1243	10.21 (1.51)	1090	10.24 (1.52)	848	10.26 (1.44)
		t_2	1732	10.19 (1.53)	1072	10.20 (1.54)	946	10.22 (1.57)	669	10.25 (1.47)
		t_3	1422	10.26 (1.53)	862	10.32 (1.56)	762	10.34 (1.58)	576	10.31 (1.47)
	Depression (HADS-D)	t_0	3019	8.50 (1.70)	1744	8.48 (1.70)	1422	8.46 (1.70)	1205	8.58 (1.79)
		t_1	1946	8.64 (1.74)	1243	8.59 (1.75)	1090	8.57 (1.74)	848	8.65 (1.78)
		t_2	1732	8.58 (1.72)	1072	8.54 (1.75)	946	8.53 (1.77)	669	8.58 (1.77)
		t_3	1422	8.58 (1.68)	862	8.55 (1.73)	762	8.51 (1.72)	576	8.59 (1.78)

			Intervention ITT 1		Intervention ITT2		Intervention AT		Control	
			n	M (SD)	n	M (SD)	n	M (SD)	n	M (SD)
**Psychosocial outcomes II**	Distress (HADS-T)	t_0	3019	18.64 (2.19)	1744	18.69 (2.20)	1422	18.69 (2.19)	1205	18.81 (2.24)
		t_1	1946	18.80 (2.14)	1243	18.80 (2.16)	1090	18.81 (2.15)	848	18.90 (2.12)
		t_2	1732	18.77 (2.09)	1072	18.74 (2.10)	946	18.75 (2.13)	669	18.83 (2.17)
		t_3	1422	18.84 (2.12)	862	18.87 (2.17)	762	18.85 (2.18)	576	18.40 (2.15)
