# Supplementary material for: Effects of telephone-based health coaching on patient-reported outcomes and health behavior change: A randomized controlled trial
Source: PLoS One. 2020 Sep 22;15(9):e0236861. doi: 10.1371/journal.pone.0236861 (PMC7508388; doi:10.1371/journal.pone.0236861)
Supplement: S4 Table — (PDF) [file pone.0236861.s004.pdf]

Supporting information 5. Model-predicted (ITT-1) estimated marginal means, their standard errors and estimated marginal differences by time (t_0, t_1, t_2, t_3), adjusted for education.

			Intervention		Control		Difference		Signifi-cance	Effect-size
			n	EMM (SE)	n	EMM (SE)	EMM difference	95%-CI	(p)	Cohen's d
Quality of life	SF-12 Mental Subscale	t_0	2625	41.22 (0.34)	1033	41.53 (0.37)	-0.31	(-0.18; 0.79)	0.217	0.05
		t_1	1728	41.09 (0.35)	760	41.17 (0.38)	-0.08	(-0.49; 0.64)	0.787	0.01
		t_2	1538	40.82 (0.36)	602	41.00 (0.40)	-0.19	(-0.43; 0.80)	0.551	0.03
		t_3	1280	41.04 (0.37)	514	41.34 (0.41)	-0.31	(-0.36; 0.97)	0.365	0.05
	SF-12 Physical Subscale	t_0	2625	36.50 (1.17)	1033	35.54 (1.19)	0.96	(-1.82; -0.11)	0.027*	-0.09
		t_1	1728	37.28 (1.18)	760	36.56 (1.20)	0.72	(-1.66; 0.21)	0.130	-0.06
		t_2	1538	37.37 (1.18)	602	36.35 (1.21)	1.02	(-2.00; -0.04)	0.042*	-0.09
		t_3	1280	36.85 (1.18)	514	35.45 (1.22)	1.40	(-2.43; -0.37)	0.008**	-0.12
	Health status (EQ5D-VAS)	t_0	2951	53.73 (1.46)	1171	52.62 (1.51)	1.11	(-2.61; 0.39)	0.148	-0.05
		t_1	1874	55.21 (1.48)	832	53.02 (1.55)	2.18	(-3.88; -0.49)	0.012*	-0.11
		t_2	1682	55.54 (1.49)	655	53.36 (1.57)	2.18	(-3.98; -0.38)	0.018*	-0.04
		t_3	1398	54.39 (1.50)	565	52.81 (1.59)	1.58	(-3.48; 0.32)	0.103	-0.07
Health behaviors	Alcohol consumption (AUDIT-C)	t_0	2575	1.97 (0.17)	1046	2.07 (0.17)	-0.09	(-0.06; 0.25)	0.215	0.04
		t_1	1789	1.77 (0.17)	824	1.90 (0.18)	-0.13	(-0.03; 0.29)	0.105	0.06
		t_2	1330	1.88 (0.17)	539	1.99 (0.18)	-0.11	(-0.06; 0.28)	0.219	0.06
		t_3	1314	1.67 (0.17)	544	1.87 (0.18)	-0.20	(0.03; 0.37)	0.022*	0.10
	Smoking	t_0	3006	1.81 (0.02)	1201	1.80 (0.02)	0.01	(-0.04; 0.02)	0.465	0.00
		t_1	1918	1.81 (0.02)	841	1.82 (0.02)	-0.00	(-0.03; 0.03)	0.980	0.00
		t_2	1616	1.83 (0.02)	622	1.82 (0.02)	0.01	(-0.04; 0.02)	0.459	0.00
		t_3	1346	1.81 (0.02)	547	1.81 (0.02)	0.00	(-0.03; 0.02)	0.773	0.00
	Physical activity (hours per week)	t_0	3061	7.24 (0.58)	1222	7.03 (0.61)	0.22	(-0.92; 0.49)	0.546	-0.02
		t_1	1962	6.70 (0.59)	858	7.49 (0.63)	-0.78	(-0.02; 1.59)	0.057	0.07
		t_2	1746	6.83 (0.60)	676	6.90 (0.65)	-0.07	(-0.80; 0.93)	0.883	0.01
		t_3	1434	6.96 (0.61)	580	6.25 (0.66)	0.71	(-1.63; 0.21)	0.130	-0.08
	Physical activity (metabolic rate per week)	t_0	3061	3535 (356)	1222	3437 (368)	98	(-457.64; 262.09)	0.594	-0.02
		t_1	1962	3408 (362)	858	3902 (378)	-494	(75.98; 911.74)	0.021*	0.09
		t_2	1746	3573 (364)	676	3534 (386)	39	(-489.35; 411.63)	0.866	-0.01
		t_3	1434	3406 (368)	580	3117 (392)	290	(-770.91; 191.08)	0.237	-0.07
	Body Mass Index (BMI) (kg/m²)	t_0	2850	28.00 (0.46)	1151	27.57 (0.47)	0.43	(-0.85; -0.00)	0.049*	-0.08
		t_1	1811	27.93 (0.46)	797	27.71 (0.47)	0.22	(-0.68; 0.24)	0.344	-0.04
		t_2	1658	28.05 (0.46)	654	27.82 (0.48)	0.24	(-0.71; 0.24)	0.325	-0.04
		t_3	1357	27.97 (0.46)	554	27.66 (0.48)	0.32	(-0.81; 0.17)	0.206	-0.06
Health behaviors	Adherence (MARS-D)	t_0	3893	23.97 (0.11)	1152	23.81 (0.11)	0.16	(-0.27; -0.04)	0.010*	-0.09
		t_1	1861	23.98 (0.11)	824	23.85 (0.12)	0.13	(-0.27; 0.00)	0.051	-0.08
		t_2	1612	24.01 (0.11)	630	23.89 (0.12)	0.12	(-0.27; 0.02)	0.099	-0.07
		t_3	1373	24.04 (0.11)	547	23.86 (0.12)	0.18	(-0.33; -0.03)	0.020*	-0.11
	Measuring blood pressure	t_0	2882	2.56 (0.06)	1146	2.47 (0.06)	0.08	(-0.17; 0.00)	0.052	-0.07
		t_1	1795	2.67 (0.06)	799	2.46 (0.07)	0.21	(-0.31; -0.11)	<.0001**	-0.18
		t_2	1601	2.59 (0.06)	621	2.43 (0.07)	0.17	(-0.27; -0.06)	0.001**	-0.15
		t_3	1354	2.56 (0.06)	549	2.50 (0.07)	0.07	(-0.17; 0.04)	0.217	-0.06
	Measuring blood sugar	t_0	2697	1.56 (0.05)	1093	1.52 (0.06)	0.03	(-0.11; 0.05)	0.424	-0.03
		t_1	1752	1.56 (0.05)	781	1.52 (0.06)	0.03	(-0.12; 0.05)	0.451	-0.03
		t_2	1563	1.58 (0.05)	617	1.53 (0.06)	0.05	(-0.14; 0.04)	0.287	-0.05
		t_3	1281	1.60 (0.05)	506	1.57 (0.06)	0.03	(-0.12; 0.06)	0.528	-0.03
	Foot monitoring self	t_0	2876	2.47 (0.06)	1150	2.46 (0.06)	0.01	(-0.09; 0.08)	0.881	-0.01
		t_1	1819	2.49 (0.06)	807	2.44 (0.07)	0.05	(-0.14; 0.05)	0.329	-0.04
		t_2	1640	2.50 (0.06)	635	2.44 (0.07)	0.07	(-0.17; 0.04)	0.213	-0.06
		t_3	1348	2.61 (0.06)	545	2.50 (0.07)	0.11	(-0.22; -0.00)	0.045*	-0.10
	Foot monitoring by physician	t_0	2625	1.64 (0.04)	1028	1.65 (0.04)	-0.01	(-0.05; 0.07)	0.777	0.01
		t_1	1686	1.63 (0.04)	721	1.61 (0.04)	0.02	(-0.09; 0.05)	0.533	-0.02
		t_2	1479	1.65 (0.04)	550	1.64 (0.04)	0.01	(-0.09; 0.06)	0.747	-0.01
		t_3	1230	1.67 (0.04)	471	1.63 (0.05)	0.04	(-0.12; 0.04)	0.294	-0.05
Psychosocial outcomes I	Patient activation (PAM)	t_0	2879	38.87 (0.35)	1156	38.50 (0.37)	0.37	(-0.78; 0.03)	0.069	-0.07
		t_1	1868	38.90 (0.36)	818	38.26 (0.38)	0.64	(-1.10; -0.19)	0.006**	-0.12
		t_2	1677	38.99 (0.36)	642	38.39 (0.38)	0.59	(-1.08; -0.11)	0.017*	-0.11
		t_3	1377	38.66 (0.36)	559	38.27 (0.39)	0.39	(-0.91; 0.12)	0.134	-0.07
	Health literacy (FCCHL)	t_0	2873	33.59 (0.36)	1156	33.10 (0.38)	0.49	(-1.00.; 0.01)	0.054	-0.07
		t_1	1857	33.71 (0.37)	812	32.87 (0.40)	0.83	(-1.41; -0.26)	0.004**	-0.12
		t_2	1626	33.60 (0.37)	639	32.39 (0.41)	1.22	(-1.82; -0.61)	<.0001**	-0.17
		t_3	1337	33.50 (0.38)	554	32.44 (0.42)	1.05	(-1.70; -0.41)	0.001**	-0.14

			Intervention		Control		Difference		Signifi-cance	Effect-size
			n	EMM (SE)	n	EMM (SE)	EMM difference	95%-CI	(p)	Cohen's d
**Psychosocial outcomes II**	Stages of Change (SOC)	t_0	2974	13.65 (0.26)	1192	13.78 (0.28)	-0.13	(-0.27; 0.53)	0.517	0.02
		t_1	1907	13.21 (0.27)	847	13.83 (0.29)	-0.62	(0.17; 1.06)	0.007**	0.11
		t_2	1700	13.21 (0.27)	661	13.79 (0.30)	-0.58	(0.11; 1.06)	0.016*	0.11
		t_3	1404	13.04 (0.27)	565	13.82 (0.31)	-0.77	(0.27; 1.27)	0.002**	0.13
	Anxiety (HADS-A)	t_0	3019	10.25 (0.14)	1205	10.31 (0.15)	-0.06	(-0.05; 0.17)	0.275	0.04
		t_1	1946	10.28 (0.14)	848	10.38 (0.15)	-0.11	(-0.02; 0.24)	0.109	0.08
		t_2	1732	10.35 (0.15)	669	10.38 (0.15)	-0.02	(-0.12; 0.16)	0.752	0.01
		t_3	1422	10.41 (0.15)	576	10.45 (0.15)	-0.04	(-0.11; 0.19)	0.588	0.03
	Depression (HADS-D)	t_0	3019	8.49 (0.07)	1205	8.61 (0.08)	-0.12	(-0.00; 0.25)	0.060	0.07
		t_1	1946	8.65 (0.08)	848	8.66 (0.08)	-0.01	(-0.14; 0.16)	0.888	0.01
		t_2	1732	8.58 (0.08)	669	8.63 (0.09)	-0.06	(-0.10; 0.22)	0.483	0.03
		t_3	1422	8.56 (0.08)	576	8.59 (0.09)	-0.03	(-0.14; 0.20)	0.735	0.02
	Distress (HADS-T)	t_0	3019	18.73 (0.16)	1205	18.91 (0.17)	-0.18	(0.03; 0.34)	0.023*	0.08
		t_1	1946	18.92 (0.16)	848	19.04 (0.17)	-0.11	(-0.07; 0.30)	0.225	0.05
		t_2	1732	18.93 (0.16)	669	19.01 (0.17)	-0.08	(-0.12; 0.28)	0.455	0.04
		t_3	1422	18.97 (0.17)	576	19.04 (0.18)	-0.07	(-0.15; 0.29)	0.528	0.03

Results are expressed as EMM(SE) =estimated marginal mean (standard error), *=significant p<.05, **=significant p<.01, t_0=Baseline, t_1=1 year, t_2= 2 years, t_3= 3 years
