# Supplementary material for: Effects of telephone-based health coaching on patient-reported outcomes and health behavior change: A randomized controlled trial
Source: PLoS One. 2020 Sep 22;15(9):e0236861. doi: 10.1371/journal.pone.0236861 (PMC7508388; doi:10.1371/journal.pone.0236861)
Supplement: S5 Table — (PDF) [file pone.0236861.s005.pdf]

Supporting information 6. Model-predicted (AT) estimated marginal means, their standard errors and estimated marginal differences by time (t_0, t_1, t_2, t_3), adjusted for education.

			**Intervention**		**Control**		**Difference**		**Significance**	**Effect-size**
			n	EMM (SE)	n	EMM (SE)	EMM difference	95%-CI	(p)	Cohen's d
**Quality of life**	SF-12 Mental Subscale	t_0	1253	41.31(0.33)	1033	41.56 (0.32)	-0.25	(-0.31; 0.81)	0.386	0.04
		t_1	971	41.25 (0.35)	760	41.20 (0.34)	0.06	(-0.69; 0.58)	0.863	-0.01
		t_2	839	40.71 (0.36)	602	41.03 (0.36)	-0.32	(-0.38; 1.01)	0.369	0.05
		t_3	695	41.16 (0.37)	514	41.38 (0.38)	-0.22	(-0.54; 0.97)	0.571	0.03
	SF-12 Physical Subscale	t_0	1253	35.62 (1.06)	1033	35.38 (1.05)	0.24	(-1.22; 0.75)	0.639	-0.02
		t_1	971	36.52 (1.07)	760	36.42 (1.06)	0.10	(-1.16; 0.95)	0.847	-0.01
		t_2	839	36.74 (1.07)	602	36.21 (1.07)	0.53	(-1.64; 0.58)	0.349	-0.05
		t_3	695	36.28 (1.08)	514	35.31 (1.08)	0.97	(-2.14; 0.20)	0.105	-0.09
	Health status (EQ5D-VAS)	t_0	1405	52.68 (1.39)	1171	52.29 (1.36)	0.39	(-2.15; 1.36)	0.661	-0.02
		t_1	1048	54.66 (1.41)	832	52.71 (1.40)	1.96	(-3.90; -0.02)	0.048*	-0.09
		t_2	919	54.93 (1.43)	655	53.04 (1.42)	1.89	(-3.94; 0.17)	0.072	-0.04
		t_3	749	53.86 (1.45)	565	52.49 (1.44)	1.36	(-3.54; 0.81)	0.219	-0.06
**Health behaviors**	Alcohol consumption (AUDIT-C)	t_0	1215	1.85 (0.14)	1046	2.08 (0.14)	-0.23	(0.06; 0.40)	0.010*	0.11
		t_1	1037	1.62 (0.14)	824	1.91 (0.14)	-0.29	(0.11; 0.47)	0.002**	0.14
		t_2	719	1.76 (0.15)	539	2.01 (0.15)	-0.24	(0.04; 0.44)	0.017*	0.12
		t_3	708	1.53 (0.15)	544	1.88 (0.15)	-0.35	(0.16; 0.55)	0.001**	0.17
	Smoking	t_0	1201	1.81 (0.02)	1201	1.79 (0.02)	0.01	(-0.04; 0.01)	0.331	0.00
		t_1	841	1.82 (0.02)	841	1.81 (0.02)	0.01	(-0.04; 0.02)	0.516	0.00
		t_2	622	1.83 (0.02)	622	1.82 (0.02)	0.02	(-0.05; 0.02)	0.291	0.00
		t_3	547	1.82 (0.02)	547	1.81 (0.02)	0.01	(-0.04; 0.03)	0.623	0.00
	Physical activity (hours per week)	t_0	1443	6.75 (0.63)	1222	6.81 (0.61)	-0.06	(-0.75; 0.86)	0.887	0.01
		t_1	1097	6.43 (0.64)	858	7.27 (0.64)	-0.84	(-0.06; 1.74)	0.068	0.08
		t_2	955	6.57 (0.65)	676	6.69 (0.65)	-0.11	(-0.85; 1.07)	0.820	0.01
		t_3	769	6.82 (0.66)	580	6.03 (0.66)	0.79	(-1.82; 0.24)	0.134	-0.09
	Physical activity (metabolic rate per week)	t_0	1443	3237 (364)	1222	3267 (359)	-31	(-379.67; 440.92)	0.884	0.01
		t_1	1097	3294 (372)	858	3730 (369)	-436	(-27.55;899.44)	0.065	0.08
		t_2	955	3347 (375)	676	3363 (376)	-15	(-481.46; 512.06)	0.952	0.00
		t_3	769	3331 (383)	580	2945 (382)	387	(-920.87; 147.78)	0.156	-0.09
	Body Mass Index (BMI) (kg/m²)	t_0	1341	28.37 (0.45)	1151	27.63 (0.44)	0.74	(-1.22; -0.26)	0.003**	-0.14
		t_1	1015	28.20 (0.45)	797	27.77 (0.45)	0.43	(-0.92; 0.07)	0.093	-0.08
		t_2	905	28.16 (0.46)	654	27.88 (0.45)	0.28	(-0.79; 0.22)	0.269	-0.05
		t_3	728	28.12 (0.46)	554	27.72 (0.45)	0.41	(-0.92; 0.11)	0.121	-0.08
	Adherence (MARS-D)	t_0	1381	24.03 (0.10)	1152	23.88 (0.10)	0.16	(-0.29; -0.02)	0.024*	-0.09
		t_1	1045	24.07 (0.10)	824	23.91 (0.10)	0.16	(-0.31; 0.01)	0.040*	-0.09
		t_2	877	24.11 (0.10)	630	23.96 (0.10)	0.16	(-0.32; 0.01)	0.059	-0.10
		t_3	737	24.11 (0.11)	547	23.93 (0.11)	0.19	(-0.36; -0.01)	0.034	-0.11
	Measuring blood pressure	t_0	1369	2.60 (0.06)	1146	2.45 (0.06)	0.15	(-0.25; -0.05)	0.003**	-0.14
		t_1	1008	2.82 (0.07)	799	2.45 (0.06)	0.38	(-0.49; -0.27)	<.0001**	-0.33
		t_2	886	2.71 (0.07)	621	2.41 (0.07)	0.29	(-0.41; -0.18)	<0.001**	-0.26
		t_3	718	2.66 (0.07)	549	2.48 (0.07)	0.18	(-0.30; -0.06)	0.004**	-0.17
	Measuring blood sugar	t_0	1285	1.53 (0.06)	1093	1.52 (0.06)	0.01	(-0.11; 0.08)	0.799	-0.01
		t_1	984	1.56 (0.07)	781	1.52 (0.06)	0.04	(-0.14; 0.06)	0.467	-0.04
		t_2	868	1.57 (0.07)	617	1.53 (0.06)	0.04	(-0.14; 0.06)	0.454	-0.04
		t_3	668	1.59 (0.07)	506	1.57 (0.06)	0.02	(-0.12; 0.09)	0.719	-0.02
	Foot monitoring self	t_0	1375	2.46 (0.08)	1150	2.44 (0.08)	0.02	(-0.12; 0.08)	0.701	-0.02
		t_1	1024	2.48 (0.08)	807	2.42 (0.08)	0.06	(-0.17; 0.05)	0.291	-0.05
		t_2	907	2.51 (0.08)	635	2.42 (0.08)	0.09	(-0.21; 0.03)	0.138	-0.08
		t_3	726	2.60 (0.08)	545	2.48 (0.08)	0.12	(-0.25; -0.00)	0.057	-0.11
	Foot monitoring by physician	t_0	1225	1.67 (0.05)	1028	1.64 (0.04)	0.03	(-0.11; 0.04)	0.381	-0.04
		t_1	947	1.67 (0.05)	721	1.61 (0.05)	0.07	(-0.15; 0.01)	0.107	-0.09
		t_2	810	1.70 (0.05)	550	1.64 (0.05)	0.06	(-0.15; 0.02)	0.153	-0.08
		t_3	662	1.72 (0.05)	471	1.63 (0.05)	0.10	(-0.19; -0.00)	0.043	-0.13
**Psychosocial outcomes I**	Patient activation (PAM)	t_0	1363	38.37 (0.30)	1156	38.42 (0.30)	-0.04	(-0.42; 0.51)	0.856	0.01
		t_1	1041	39.06 (0.31)	818	38.18 (0.31)	0.88	(-1.40; -0.36)	0.001**	-0.16
		t_2	918	38.96 (0.32)	642	38.32 (0.32)	0.65	(-1.20; -0.10)	0.021*	-0.12
		t_3	747	38.52 (0.33)	559	38.19 (0.32)	0.33	(-0.92; 0.25)	0.260	-0.06
	Health literacy (FCCHL)	t_0	1372	33.21 (0.36)	1156	33.09 (0.35)	0.13	(-0.70; 0.45)	0.667	-0.02
		t_1	1045	34.00 (0.37)	812	32.86 (0.36)	1.14	(-1.77; -0.50)	0.001**	-0.16
		t_2	895	34.05 (0.38)	639	32.38 (0.38)	1.66	(-2.34; -0.99)	<.0001**	-0.23
		t_3	716	34.03 (0.39)	554	32.44 (0.38)	1.59	(-2.31; -0.87)	<0.001**	-0.21

			Intervention		Control		Difference		Signifi-cance	Effect-size
			n	EMM (SE)	n	EMM (SE)	EMM difference	95%-CI	(p)	Cohen's d
**Psychosocial outcomes II**	Stages of Change (SOC)	t_0	1411	13.32 (0.32)	1192	13.60 (0.31)	-0.28	(-0.18; 0.74)	0.240	0.05
		t_1	1070	12.65 (0.33)	847	13.64 (0.32)	-1.00	(0.49; 1.49)	<0.001**	0.17
		t_2	931	12.60 (0.33)	661	13.60 (0.33)	-1.01	(0.48; 1.54)	<0.001**	0.18
		t_3	759	12.62 (0.34)	565	13.63 (0.33)	-1.01	(0.45; 1.57)	<0.001**	0.17
	Anxiety (HADS-A)	t_0	1422	10.33 (0.13)	1205	10.27 (0.13)	-0.05	(-0.18; 0.08)	0.421	-0.03
		t_1	1090	10.30 (0.14)	848	10.35 (0.14)	-0.05	(-0.09; 0.20)	0.483	0.03
		t_2	946	10.33 (0.14)	669	10.34 (0.14)	-0.01	(-0.15; 0.17)	0.894	0.01
		t_3	762	10.48 (0.14)	576	10.42 (0.14)	0.06	(-0.23; 0.11)	0.514	-0.04
	Depression (HADS-D)	t_0	1422	8.47 (0.09)	1205	8.61 (0.09)	-0.14	(-0.01; 0.29)	0.065	0.08
		t_1	1090	8.60 (0.09)	848	8.67 (0.09)	-0.07	(-0.10; 0.24)	0.406	0.04
		t_2	946	8.56 (0.10)	669	8.64 (0.10)	-0.08	(-0.10; 0.26)	0.384	0.05
		t_3	762	8.51 (0.10)	576	8.59 (0.10)	-0.08	(-0.12; 0.27)	0.424	0.04
	Distress (HADS-T)	t_0	1422	18.80 (0.17)	1205	18.88 (0.17)	-0.09	(-0.10; 0.27)	0.356	0.04
		t_1	1090	18.89 (0.17)	848	19.01 (0.17)	-0.12	(-0.09; 0.33)	0.259	0.06
		t_2	946	18.89 (0.17)	669	18.98 (0.18)	-0.09	(-0.14; 0.32)	0.425	0.04
		t_3	762	18.99 (0.18)	576	19.01 (0.18)	-0.02	(-0.22; 0.27)	0.857	0.01

Results are expressed as EMM(SE) =estimated marginal mean (standard error), *=significant p<.05, **=significant p<.01, t_0=Baseline, t_1=1 year, t_2= 2 years, t_3= 3 years.
