# Supplementary material for: Effects of telephone-based health coaching on patient-reported outcomes and health behavior change: A randomized controlled trial
Source: PLoS One. 2020 Sep 22;15(9):e0236861. doi: 10.1371/journal.pone.0236861 (PMC7508388; doi:10.1371/journal.pone.0236861)
Supplement: S6 Table — (PDF) [file pone.0236861.s006.pdf]

S7 Table. Model-predicted (time x group x campaign; ITT-2) estimated marginal means, their standard errors and estimated marginal differences by time (t0, t1, t2, t3), adjusted for education and age for "measuring blood sugar" and "foot monitoring by physician".

		chronic campaign 1				chronic campaign 2				heart failure				mental health			
		Intervention		Control		Intervention		Control		Intervention		Control		Intervention		Control	
		n	EMM (SE)	n	EMM (SE)	n	EMM (SE)	n	EMM (SE)	n	EMM (SE)	n	EMM (SE)	n	EMM (SE)	n	EMM (SE)
Measuring blood sugar	t_0	330	1.62 (0.07)		1.70 (0.07)	972	1.84 (0.05)		1.76 (0.05)	160	1.77 (0.10)		2.14 (0.11)	71	1.08 (0.16)		1.08 (0.16)
	t_1	237	1.67 (0.08)	169	1.70 (0.08)	717	1.88 (0.05)	510	1.76 (0.05)	105	1.72 (0.10)	70	2.01 (0.12)	35	1.14 (0.17)	27	1.24 (0.17)
	t_2	209	1.80 (0.08)	138	1.67 (0.08)	638	1.86 (0.05)	398	1.79 (0.05)	87	1.72 (0.11)	52	1.98 (0.12)	31	1.03 (0.17)	24	1.21 (0.18)
	t_3	158	1.80 (0.08)	125	1.76 (0.08)	494	1.87 (0.05)	319	1.80 (0.05)	73	1.93 (0.11)	38	1.94 (0.13)	19	1.01 (0.19)	20	1.47 (0.18)
Foot monitoring by physician	t_0	302	1.72 (0.55)		1.78 (0.06)	936	1.87 (0.03)		1.81 (0.04)	165	1.79 (0.07)		1.84 (0.09)	56	1.36 (0.13)		1.29 (0.12)
	t_1	230	1.74 (0.06)		1.69 (0.07)	704	1.86 (0.04)		1.77 (0.04)	103	1.82 (0.09)		1.93 (0.10)	25	1.27 (0.18)		1.34 (0.16)
	t_2	199	1.78 (0.06)	111	1.75 (0.07)	606	1.87 (0.04)	370	1.81 (0.04)	85	1.89 (0.09)	44	1.71 (0.11)	19	1.29 (0.19)	20	1.36 (0.16)
	t_3	161	1.80 (0.07)	99	1.75 (0.08)	490	1.90 (0.04)	316	1.79 (0.04)	72	1.77 (0.10)	35	1.87 (0.12)	13	1.21 (0.21)	16	1.44 (0.18)

Results are expressed as EMM(SE) =estimated marginal mean (standard error)
