# Supplementary material for: Effects of telephone-based health coaching on patient-reported outcomes and health behavior change: A randomized controlled trial
Source: PLoS One. 2020 Sep 22;15(9):e0236861. doi: 10.1371/journal.pone.0236861 (PMC7508388; doi:10.1371/journal.pone.0236861)
Supplement: S1 File — (PDF) [file pone.0236861.s007.pdf]

Dwinger et al. Trials 2013, 14:337
http://www.trialsjournal.com/content/14/1/337

TRIALS

STUDY PROTOCOL Open Access

# Telephone-based health coaching for chronically ill patients: study protocol for a randomized controlled trial

Sarah Dwinger[1*†], Jörg Dirmaier[1†], Lutz Herbarth[2], Hans-Helmut König[3], Matthias Eckardt[3], Levente Kriston[1], Isaac Bermejo[4] and Martin Härter[1]

## Abstract

**Background:** The rising prevalence of chronic conditions constitutes a major burden for patients and healthcare systems and is predicted to increase in the upcoming decades. Improving the self-management skills of patients is a strategy to steer against this burden. This could lead to better outcomes and lower healthcare costs. Health coaching is one method for enhancing the self-management of patients and can be delivered by phone. The effects of telephone-based health coaching are promising, but still inconclusive. Economic evaluations and studies examining the transferability of effects to different healthcare systems are still rare. Aim of this study is to evaluate telephone-based health coaching for chronically ill patients in Germany.

**Methods/Design:** The study is a prospective randomized controlled trial comparing the effects of telephone-based health coaching with usual care during a 4-year time period. Data are collected at baseline and after 12, 24 and 36 months. Patients are selected based on one of the following chronic conditions: diabetes, coronary artery disease, asthma, hypertension, heart failure, COPD, chronic depression or schizophrenia. The health coaching intervention is carried out by trained nurses employed by a German statutory health insurance. The frequency and the topics of the health coaching are manual-based but tailored to the patients' needs and medical condition, following the concepts of motivational interviewing, shared decision-making and evidence-based-medicine. Approximately 12,000 insurants will be enrolled and randomized into intervention and control groups. Primary outcome is the time until hospital readmission within two years after enrolling in the health coaching, assessed by routine data. Secondary outcomes are patient-reported outcomes like changes in quality of life, depression and anxiety and clinical values assessed with questionnaires. Additional secondary outcomes are further economic evaluations like health service use as well as costs and hospital readmission rates. The statistical analyses includes intention-to-treat and as-treated principles. The recruitment will be completed in September 2014.

**Discussion:** This study will provide evidence regarding economic and clinical effects of telephone-delivered health coaching. Additionally, this study will show whether health coaching is an adequate option for the German healthcare system to address the growing burden of chronic diseases.

**Trial registration:** German Clinical Trials Register (Deutsches Register Klinischer Studien; DRKS) DRKS00000584.

**Keywords:** Chronic conditions, Health coaching, Telephone-based, Economic evaluation, Routine data

* Correspondence: s.dwinger@uke.de
†Equal contributors
[1]Department of Medical Psychology, University Medical Center Hamburg-Eppendorf, Martinistr 52, Hamburg 20246, Germany
Full list of author information is available at the end of the article

BioMed Central

© 2013 Dwinger et al.; licensee BioMed Central Ltd. This is an open access article distributed under the terms of the Creative Commons Attribution License (http://creativecommons.org/licenses/by/2.0), which permits unrestricted use, distribution, and reproduction in any medium, provided the original work is properly cited.

## Background

### Chronic conditions

The leading causes of death and disability are chronic diseases such as, for example, diabetes mellitus, heart diseases or cancer [1]. Because of the rapid aging of the population, longer life expectancy, and medical progress, chronic diseases are expected to cause over three-quarters of all deaths by 2030 [2]. Additionally, an increasing percentage of patients with chronic conditions are multimorbid [3]. Chronic conditions are a burden for the healthcare system and also for the patients and their families. People with a chronic disease have a reduced potential life span and reduced quality of life [4].

### Handling chronic conditions

Due to the high prevalence of chronic diseases, it is important to find strategies to steer against the increasing burden for patients as well as society. One attempt to handle this burden is to increase the self-management of patients since the course of chronic conditions can be influenced significantly by health behavior changes. Therefore, chronic diseases are at least partially considered to be preventable or modifiable [5]. If patients change to a healthier lifestyle by stopping smoking, drinking less alcohol, doing more exercise or changing dietary behavior, they can actively contribute to preventing and controlling complications from chronic diseases [6]. Active patients show greater adherence, know more about their disease and can achieve improved health [7]. Active involvement and improved self-management skills of chronically ill patients may even reduce healthcare costs [8].

One specific method to improve self-management and patient involvement is health coaching. Aims of health coaching are to improve adherence to health behaviors and to support lifestyle changes in order to prevent a negative course of chronic illness [9]. Besides the education of patients by providing evidence-based information, health coaching includes communication styles that increase the motivation to change risky health behaviors. Common strategies of effective coaching programs are goal setting strategies, motivational interviewing, collaboration with healthcare providers [10] and shared decision-making [11]. Health coaching is tailored to the patients' knowledge and needs. It is an addition to the medical consultation by preparing the patient before the consultation and clarifying relevant questions afterwards [12].

### Telephone-based health coaching

There are many ways to deliver health coaching; one way is coaching by phone, which allows reaching even rural areas and impaired patients. The increasing number of studies that evaluate telephone-based health coaching (TBHC) for chronically ill patients shows promising but also conflicting results. For example, participants of TBHC have reduced diabetes symptoms as well as lowering depressive symptoms [13] and gaining a better total cholesterol and LDL-value [14]. It seems that TBHC has the potential to reduce the rate of coronary events, like myocardial infarction, coronary artery bypass graft and cardiovascular death [15]. However, there are also some studies showing that there are no improvements regarding targeted clinical outcomes [16]. Furthermore, TBHC can help participants change health behavior [17], enhance self-rated health [18], social functioning, self-efficacy, patient activation and perceived health [13,17].

Economic evaluations are rare and still contradictory to some extent. Studies show that TBHC can reduce the number of contacts to the general practitioner [19], hospitalization rates and healthcare costs [11]. However, other studies show only modest effects on hospital admissions or emergency room visits. Also, these studies show no reduction of utilization and costs accompanied by high costs for the admission of the coaching [20,21].

In summary, the results of studies evaluating TBHC are ambiguous. The clearest effects can be observed with psychosocial and self-reported outcomes, like self-efficacy and perceived health. Considering economic effects and clinical outcomes, results of empirical studies are still inconsistent.

In Germany, health coaching is an innovative approach, and the number of studies evaluating TBHC in Germany is very limited. Long-term effects and cost-effectiveness have not been analyzed yet. However, the patients' acceptance of the intervention is good [22-24]. The TBHC used in this study is based on a TBHC service developed by Health Dialog [11,25] and was implemented by Kaufmännische Krankenkasse Hannover (KKH), a statutory health insurance, in 2007. The coaching was adapted to the German patients' requirements after assessing the needs in a previous study [22].

The German healthcare system is contribution-financed and based on self-government and solidarity. The healthcare delivery is not centralized but provided by a complex network of public bodies at law and a large number of independent regional and local bodies. Because the German healthcare system differs significantly from others, the transferability of results of international studies still needs to be verified.

### Objectives

The objective of the trial is to evaluate the effectiveness of TBHC for patients of a health insurance fund against usual care regarding its impact on healthcare costs and patient-reported outcomes. The primary hypothesis is that TBHC will extend the time period until hospital readmission. Secondly, we hypothesize that TBHC will reduce healthcare costs and hospital readmission rates.

Furthermore, the positive impact of TBHC on patient-reported outcomes like quality of life (QoL), depression and anxiety, health behavior, health literacy, patient activation and the evaluation of TBHC by participants will be evaluated. We also expect TBHC to increase quality of life and decrease psychological distress such as depression or anxiety.

## Methods/Design

### Study design

The study is a prospective randomized controlled trial (RCT) comparing TBHC with patients in usual care during a 4-year time period. There are three follow-up measures; in addition to the baseline measure ($T_0$) the data will be assessed again at 12 months ($T_1$), 24 months ($T_2$) and 36 months ($T_3$). The length of the coaching will be tailored to the needs of the patient, but will not exceed two years.

The studies comply with the Helsinki Declaration 2008. The ethics approval was granted by the Hamburg Medical Chamber Ethics Committee.

### Sample procedures

#### *Inclusion criteria for patients*

Participants should be at least 18 years old and insurants of the KKH statutory health insurance. Moreover, included patients will need to have one or more diagnoses of the following: diabetes, coronary artery disease, asthma, hypertension, heart failure, chronic obstructive pulmonary disease (COPD), chronic depression or schizophrenia. Additional inclusion criteria will be used for each target disease. For example, for patients with type 2 diabetes, hypertension or coronary artery diseases, a risk score for hospital readmission will be calculated. Based on various variables from routine data a logistic regression model was designed to predict hospital readmission using SPSS 19. Those variables are, for example, healthcare costs, international classification of diseases (ICD)-diagnoses, age and gender. This model has been validated by analyzing the receiver operating characteristic curve (ROC) [26] and the positive predictive value [27] and showed valid predictions. If the calculated risk for hospital readmission within the next year is higher than 50%, the person will be included in the study. Patients will be excluded from the study if they have insufficient German language skills, are hard of hearing or are not able to read or use a phone.

### Study procedures

The eligible insurants will be randomized to the intervention and control group. After randomization, members of the intervention group will receive an invitation to take part in the TBHC and an acquisition call. If they send back the confirmation of participation, they will be included in the study as participants. In case they will not send back the required confirmation, insurants will be grouped as decliners. The control group will receive no invitation.

After completion of the treatment allocation, envelopes will be sent out to the members of the three groups. The first mailing consists of a cover letter, an explanation of the study, information regarding privacy, an informed consent and the questionnaire itself. Six weeks after the first letter, a reminder will be sent out with the same content. In order to limit expenses, just 50% of the decliners who will be randomly selected will receive an envelope (Figure 1).

### Treatment allocation

To ensure comparability between the intervention and the control group, a stratified random allocation design will be used based on sociodemographic values with a 5:1 allocation ratio to intervention and control group. The randomized intervention group consists of participants and decliners. The decliners will not receive any TBHC. However, they will have the opportunity to opt in to the TBHC via a telephone number printed on the questionnaires. In that case, they will be excluded from the study.

Once the patients are assigned to one of the three groups there will be no blinding of coaches, patients or researchers and their assistants. Nevertheless, the coaches will not know who is responding in the study. The questionnaires are pseudonymized.

#### *Power calculation*

Calculation of the target sample size was based on the patient reported outcomes collected by questionnaire. In order to achieve a power of at least 95% at a type I error rate of $\alpha = 5\%$ in a two-sided test accounting for the unbalanced group allocation, a minimum of 1,670 patients are needed to be able to detect a small standardized mean difference in group comparisons (Cohen's d of 0.2) [28] at $T_2$ following the intention-to-treat principle. Assuming a response rate between 18% and 40% and drop-out rates between 10% and 82% depending on group and phase, a target sample size of approximately 9,000 patients was identified who should be invited to the questionnaire survey. Assuming that around 65% of the invited patients will decline participation and half of them will be followed up, a total of 12,000 patients were targeted (see Figure 1). Because the primary outcome 'time until hospital readmission' is collected from routine data available for the total sample of 12,000 patients from the KKH statutory health insurance, the primary analysis is sufficiently powered to detect even very small effects with a high probability (low type II error).

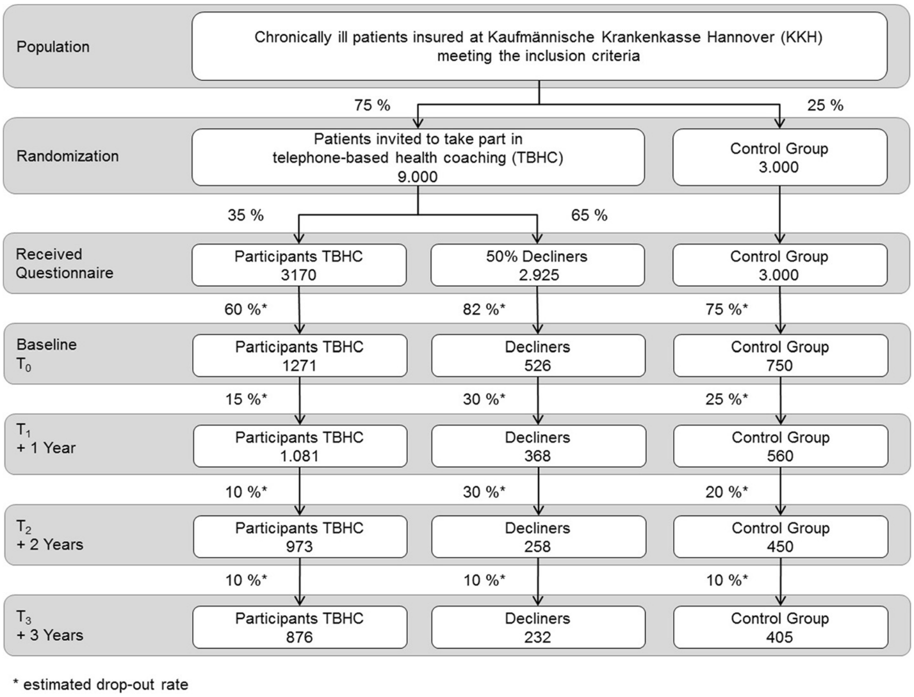


**Figure 1 Expected participant flow for patient-reported outcomes.**

## Description of the intervention and control condition

### *Intervention condition*

The TBHC will follow the concepts of motivational interviewing techniques, individual, collaborative goal setting and shared decision-making. The coach will lead the conversation, but will try to follow a non-directive approach. There will be a minimum frequency defined as one telephone contact every six weeks. The TBHC will have a maximum duration of two years. The intervention will be tailored to chronic diseases that are in need of similar self-management strategies: 1) asthma and COPD, 2) type 2 diabetes, hypertension and coronary artery disease, 3) heart failure and 4) depression and schizophrenia. Each of these disease-specific implementations of the TBHC intervention will have its own treatment manual. These manuals will include information about the inclusion criteria, the scientific background of the campaign and its key contents. All components of the campaign such as the coaching process will be described as well. For example, there will be instructions for specific coaching situations and main topics (for example, diet, immunization and foot control in diabetes and complementary medicine). Even though these instructions exist, the coaching will be tailored to the patients' needs and his individual medical condition. Additionally, the coaches will be supported by a web library and a software program. The web library will provide health information to the coaches to ensure that the information given is evidence-based and up-to-date (for example, by referring to actual clinical practice guidelines). The software contains available printed information material such as education leaflets for specific conditions, medication plans and weight-control tables that can be sent to the participants by mail. The software also offers a tool to document the process, individual goals, medication, clinical parameters (like $HbA_{1c}$ or blood pressure) and diagnoses.

The coaches will be experienced nurses as well as one nutrition scientist and two coaches who have special expertise with psychiatric patients. They will have been especially trained to carry out the coaching by staff directly qualified by Health Dialog, the company that developed this TBHC in the United States. The coaches will be located in two areas in Germany: one team is operating from Munich and one from Halle/Saale. The coaches will be supervised two to three times a year by two experienced supervisors from the project management group (MH, IB).

### *Control condition*

The control group will receive no coaching (treatment as usual). It is possible that the control group will receive the same written information material as the intervention group through other employees of the insurance.

## Outcome assessment

### *Primary outcome*

The primary outcome is the time from enrollment until hospital readmission within two years. Time until

hospital readmission will be assessed using routine health insurance data of the KKH that records the date of enrollment in the study as well as the dates of hospital stay.

The routine data will be collected and provided by the statutory health insurance. They are generated during the everyday healthcare business and show the actual healthcare situation of the insurant. Every contact within the healthcare system is represented: doctor and hospital visits with ICD code and Operationen- und Prozedurenschlüssel (OPS) code, the German equivalent to the American procedure coding system (PCS), incapacity to work, the medication and much more. The data are not influenced by non-response or recall-bias as they are assessed directly at the healthcare provider. Routine data depicts accountable variables, but not clinical parameters. The validity of routine data in Germany is not proven, yet studies show that the ICD diagnoses are valid in about 97% for heart failure [29,30]. The routine data are read out by the KKH and securely delivered with pseudonymized codes. The outcomes based on routine data include data of the whole randomized sample of approximately 12,000 insurants.

#### *Secondary outcomes*

The secondary outcomes include further economic evaluation and the evaluation of patient-reported outcomes.

Secondary outcomes for the health economic evaluation refer to health service use and costs. These include hospital care (hospital admission rates, hospital days and hospital costs) as well as outpatient physician and non-physician services, rehabilitation services and medication. Furthermore, frequency and duration of inability to work as well as mortality will be assessed. Assessment of these outcomes is based on routine data from the KKH.

Moreover, several patient-reported outcomes are used as secondary outcomes, for example, changes in the quality of life (QoL), depression and anxiety, health related behavior and changes in clinical outcomes. The used instruments are mostly validated translations of internationally well-known and standardized instruments with satisfying validity and reliability. The QoL is assessed through the 'Short Form 12 Health Survey' (SF-12) [31] and depression and anxiety are assessed through the 'Hospital Anxiety and Depression Scale' (HADS) [32]. The change of health related behavior is assessed with the 'Alcohol Consumption Questions of the Alcohol Use Disorders Identification Test' (AUDIT-C) [33]. Additionally, a self-developed instrument assesses the amount of smoking, the German version of the 'Medication Adherence Report Scale' (MARS-D) measures medication adherence [34], the amount of exercise is assessed by the 'Freiburg Questionnaire for Physical Activity' (FFKA) [35] and a newly developed instrument assesses the stages of change. Clinical parameters like glycosylated hemoglobin ($HbA_{1c}$) and blood pressure are measured with self-developed items.

### Statistical analysis

Following the intention-to-treat approach we will include all randomized participants in the primary analyses in order to avoid biases such as non-random attrition of participants. As many of the randomized participants decline to take part in the TBHC we run a second intention-to-treat approach, testing the participants versus the control group. Additionally we will perform a sensitivity analysis following the as-treated approach including only participants that have received a minimum of five coaching calls.

Therefore, three different analyses will be performed:

1) Intention-to-treat 1: comparing the randomized intervention group (including decliners) versus the control group.
2) Intention-to-treat 2: comparing those who took part in the intervention versus the control group (excluding the decliners).
3) As-treated: comparing participants who received a minimum of five calls versus the control group.

In order to enhance the comparability of intervention group and control group in the health economic outcomes for analyses 2 and 3, propensity score matching (PSM) will be used to control potential confounding. Routine data from the 12-month period preceding recruitment will be used to match individuals in the intervention group with controls by means of propensity scores. Formally, the propensity score is a person's probability of being assigned to the intervention group given a set of observed covariates. The covariates in the propensity score model will be based on routine data: for example, age, sex, ICD diagnoses, number of days spent in hospital and healthcare costs in the preceding year. The predicted probabilities of unconditional logistic regression models with 'treatment' (intervention versus controls) as dependent variable and the described set of covariates are the basis for the subsequent matching of both groups.

For the primary outcome 'time until hospital readmission' we will use the hazard ratio from a proportional hazard model. Service use and costs will be analyzed by logistic and linear random effects regression models using bootstrapped standard errors.

The secondary outcomes that are patient reported will include different scales, instruments and items with various scale levels. For the analyses of nominal scaled outcomes we will use chi-square tests. Ordinal and interval scaled outcomes will be analyzed using ANOVA

and ANCOVA. If there are significant differences between the groups we will adjust for covariates by using logistic regression. Results with an alpha error rate $\alpha \leq 0.05$ were mostly considered statistically significant. As we are testing more than one comparison we need a Bonferroni correction to control the familywise error rate.

Missing data will be addressed by at least two procedures (for example, complete case analyses, imputation by expectation-maximization, imputation by last observation carried forward) depending on the outcome and the missing data structure in order to test robustness of the findings.

## Discussion

This study is a 4-year RCT that examines the economic and psychosocial evaluation of a telephone-based health coaching. The evaluated intervention is a health coaching delivered by phone by specially trained nurses. It focuses on lifestyle change using motivational interviewing techniques combined with individual goal setting. The coaching is manualized and computer supported.

As there are just few publications about the health economic evaluation of telephone-based health coaching (TBHC) programs, and especially few about TBHC in the German healthcare system, this trial can fill the existing gap. Our findings will be relevant for future health coaching programs as potential cost savings are interesting for statutory health insurances.

Nevertheless, there are some limitations to this study: Randomization took place before informed consent was obtained from participants. As participants and decliners are likely to be different, this may cause bias with respect to the groups compared in the intention-to-treat-2 and the as-treated analysis. Although we apply advanced statistical methods, potential confounding cannot be ruled out completely. Another limitation could be the unsure validity of the routine data, but an extensive literature research revealed that the quality of routine data in Germany is good [29]. The generalizability of the secondary patient reported outcomes may be limited by social desirability, because the statutory health insurance is sending out the questionnaires. To prevent this bias we assured the insurants in the cover letter that non-response will definitely have no negative consequences. Another possible limitation is the quality of the items questioning the clinical parameters like $HbA_{1c}$. They are patient reported on an ordinal scale, which means they are prone to be inexact. To approach this problem we take advantage of the opportunity to use the software data available for the participant group as well.

Since the amount of studies evaluating psychological and economic outcomes of health coaching interventions are rare, our study will provide evidence, which is needed for different healthcare bodies. The study's outcome will help determine whether TBHC is a promising tool to enhance the self-management of chronically ill patients and thus reduce costs, increase quality of life and change clinical parameters.

## Trial status

Recruitment of participants started in May 2010 and is expected to end in September 2014.

### Abbreviations

ANOVA: Analysis of variance; ANCOVA: Analysis of covariance; AUDIT-C: Alcohol consumption questions of the alcohol use disorders identification test; COPD: Chronic obstructive pulmonary disease; DMP: Disease management program; FFKA: Freiburg questionnaire for physical activity; HADS: Hospital anxiety and depression scale; ICD: International classification of diseases; $HbA_{1c}$: Glycosylated hemoglobin (hemoglobin A1c); KKH: Kaufmännische Krankenkasse Hannover; MARS-D: Medication adherence report scale; OPS: Operationen- und Prozedurenschlüssel; PCS: Procedure coding system; PSM: Propensity score matching; QoL: Quality of life; RCT: Randomized controlled trial; ROC: Receiver operating characteristic curve; PSM: Propensity score matching; TBHC: Telephone-based health coaching.

### Competing interests

The study is funded by Kaufmännische Krankenkasse Hannover (KKH). One of the co-authors (LH) is employed by KKH. The authors declare that they have no competing interests.

### Authors' contributions

All authors have made substantial contributions to this manuscript and the final version is a result of several discussions and team work among the authors. MH, IB, JD and LK worked together with the study design. SD made the first draft of the paper and JD, LH and MH revised and made substantial contribution to the manuscript. HHK and ME developed the health economic part. All authors read and approved the final manuscript.

### Acknowledgements

We would like to acknowledge Kaufmännische Krankenkasse Hannover for financing the project.

### Author details

[1]Department of Medical Psychology, University Medical Center Hamburg-Eppendorf, Martinistr 52, Hamburg 20246, Germany. [2]KKH Kaufmännische Krankenkasse, Karl-Wiechert-Allee 61, Hannover 30625, Germany. [3]Department of Health Economics and Health Services Research, University Medical Center Hamburg-Eppendorf, Martinistr 52, Hamburg 20246, Germany. [4]Celenus Kliniken, Moltkestraße 27, Offenburg 77654, Germany.

Received: 3 July 2013 Accepted: 1 October 2013
Published: 17 October 2013

### References

1. WHO: *The European health report 2009: health and health systems.* Copenhagen: World Health Organization, Regional Office for Europe; 2009.
2. Mathers CD, Loncar D: **Projections of global mortality and burden of disease from 2002 to 2030.** *PLoS Med* 2006, **3:**e442.
3. Boyd CM, Fortin M: **Future of multimorbidity research: how should understanding of multimorbidity inform health system design?** *Public Health Rev* 2010, **32:**451–474.
4. Jia H, Zack MM, Thompson WW: **The effects of diabetes, hypertension, asthma, heart disease, and stroke on quality-adjusted life expectancy.** *Value Health* 2013, **16:**140–147.
5. Gardener H, Wright CB, Gu Y, Demmer RT, Boden-Albala B, Elkind MS, Sacco RL, Scarmeas N: **Mediterranean-style diet and risk of ischemic stroke, myocardial infarction, and vascular death: the Northern Manhattan Study.** *Am J Clin Nutr* 2011, **94:**1458–1464.
6. Shrestha P, Ghimire L: **A review about the effect of life style modification on diabetes and quality of life.** *Glob J Health Sci* 2012, **4:**185–190.

7. Coulter A, Ellins J: Effectiveness of strategies for informing, educating, and involving patients. *BMJ* 2007, 335:24–27.
8. Hibbard JH, Greene J, Overton V: Patients with lower activation associated with higher costs; delivery systems should know their patients' 'scores'. *Health Aff* 2013, 32:216–222.
9. Huffman MH: Health coaching: a fresh, new approach to improve quality outcomes and compliance for patients with chronic conditions. *Home Healthc Nurse* 2009, 27:490–496. quiz 496-498.
10. Olsen JM, Nesbitt BJ: Health coaching to improve healthy lifestyle behaviors: an integrative review. *Am J Health Promot* 2010, 25:e1–e12.
11. Wennberg DE, Marr A, Lang L, O'Malley S, Bennett G: A randomized trial of a telephone care-management strategy. *N Engl J Med* 2010, 363:1245–1255.
12. O'Connor AM, Stacey D, Legare F: Coaching to support patients in making decisions. *BMJ* 2008, 336:228–229.
13. Sacco WP, Malone JI, Morrison AD, Friedman A, Wells K: Effect of a brief, regular telephone intervention by paraprofessionals for type 2 diabetes. *J Behav Med* 2009, 32:349–359.
14. Vale M, Jelinek M, Best J, Santamaria J: Coaching patients with coronary heart disease to achieve to target cholesterol: a method to bridge the gap between evidence-based medicine and the "real world" - randomized controlled trial. *J Clin Epidemiol* 2002, 55:245–252.
15. Lisspers J, Sundin Ö, Öhman A, Horfman-Bang C, Rydén L, Nygren A: Long-term effects of lifestyle behavior change in coronary artery disease: effects on recurrent coronary events after percutaneous coronary intervention. *Health Psychol* 2005, 24:41–48.
16. Patja K, Absetz P, Auvinen A, Tokola K, Kyto J, Oksman E, Kuronen R, Ovaska T, Harno K, Nenonen M, Wiklund T, Kettunen R, Talja M: Health coaching by telephony to support self-care in chronic diseases: clinical outcomes from The TERVA randomized controlled trial. *BMC Health Serv Res* 2012, 12:147.
17. Linden A, Butterworth SW, Prochaska JO: Motivational interviewing-based health coaching as a chronic care intervention. *J Eval Clin Pract* 2010, 16:166–174.
18. Bambauer KZ, Aupont O, Stone PH, Locke SE, Mullan MG, Colagiovanni J, McLaughlin TJ: The effect of a telephone counseling intervention on self-rated health of cardiac patients. *Psychosom Med* 2005, 67:539–545.
19. Browning C, Thomas S: Six-month outcome data for the Good Life Club project: an outcomes study of diabetes self-management. *Aust J Prim Health* 2003, 9:192–198.
20. McCall N, Cromwell J: Results of the medicare health support disease-management pilot program. *N Engl J Med* 2011, 365:1704–1712.
21. Lin WC, Chien HL, Willis G, O'Connell E, Rennie KS, Bottella HM, Ferris TG: The effect of a telephone-based health coaching disease management program on Medicaid members with chronic conditions. *Med Care* 2012, 50:91–98.
22. Härter M, Dwinger S, Seebauer L, Simon D, Herbarth L, Siegmund-Schultze E, Temmert D, Bermejo I, Dirmaier J: Evaluation of telephone health coaching of German health insurants with chronic conditions. *Health Educ J* 2013, 72:622–634.
23. Bohme S, Geiser C, Muhlenhoff T, Holtmann J, Renneberg B: Telephone counseling for patients with chronic heart failure: results of an evaluation study. *Int J Behav Med* 2012, 19:288–297.
24. Seebauer L, Simon D, Bermejo I, Herbarth L, Siegmund-Schultze E, Temmert D, Schlegel D, Härter M: Attitudes of chronically-ill patients to telephone health coaching. *Gesundheitswesen* 2011, 73:430–437.
25. *Health Dialog.* www.healthdialog.com.
26. McClish DK: Analyzing a portion of the ROC curve. *Med Decis Making* 1989, 9:190–195.
27. Altman DG, Bland JM: Statistics notes: diagnostic tests 2: predictive values. *BMJ* 1994, 309:102.
28. Cohen J: *Statistical Power Analysis for the Behavioral Sciences.* 2nd edition. Hillsdale, NJ: Lawrence Erlbaum Associates; 1988.
29. Schubert I, Ihle P, Koster I: Internal confirmation of diagnoses in routine statutory health insurance data: concept with examples and case definitions. *Gesundheitswesen* 2010, 72:316–322.
30. Hoffmann F, Pfannkuche M, Glaeske G: Wie häufig sind Verordnungs- und Abgabedatum von Arzneimitteln in Routinedaten korrekt erfasst? *Bundesgesundheitsblatt Gesundheitsforschung Gesundheitsschutz* 2007, 50:1418–1423.
31. Bullinger M, Kirchberger I: *SF-36-Fragebogen zum Gesundheitszustand: Handanweisung.* Hogrefe, Verlag für Psychologie: Göttingen; 1998.
32. Herrmann-Lingen C, Buss U, Snaith RP: *HADS-D: hospitality anxiety and depression scale: deutsche version: ein fragebogen zur erfassung von angst und depressivität in der somatischen medizin.* Bern: Huber; 2005.
33. Bush K, Kivlahan DR, McDonell MB, Fihn SD, Bradley KA: The AUDIT alcohol consumption questions (AUDIT-C): an effective brief screening test for problem drinking. Ambulatory care quality improvement project (ACQUIP). Alcohol use disorders identification test. *Arch Intern Med* 1998, 158:1789–1795.
34. Mahler C, Hermann K, Horne R, Ludt S, Haefeli WE, Szecsenyi J, Jank S: Assessing reported adherence to pharmacological treatment recommendations. Translation and evaluation of the Medication Adherence Report Scale (MARS) in Germany. *J Eval Clin Pract* 2010, 16:574–579.
35. Frey I, Berg A, Grathwohl D, Keul J: Freiburg Questionnaire of physical activity–development, evaluation and application. *Soz Praventivmed* 1999, 44:55–64.

doi:10.1186/1745-6215-14-337
**Cite this article as:** Dwinger *et al.*: Telephone-based health coaching for chronically ill patients: study protocol for a randomized controlled trial. *Trials* 2013 14:337.

**Submit your next manuscript to BioMed Central and take full advantage of:**

- Convenient online submission
- Thorough peer review
- No space constraints or color figure charges
- Immediate publication on acceptance
- Inclusion in PubMed, CAS, Scopus and Google Scholar
- Research which is freely available for redistribution

Submit your manuscript at
www.biomedcentral.com/submit

BioMed Central
