# Supplementary material for: Effects of telephone-based health coaching on patient-reported outcomes and health behavior change: A randomized controlled trial
Source: PLoS One. 2020 Sep 22;15(9):e0236861. doi: 10.1371/journal.pone.0236861 (PMC7508388; doi:10.1371/journal.pone.0236861)
Supplement: S2 File — (PDF) [file pone.0236861.s008.pdf]

Supporting Information 8: Study protocol for ethics committee in German and partly translated into English (submitted prior to patient inclusion)

Study protocol for the study „Evaluation of a telephone-based health coaching for chronic patients"

Scientific question

Topic of the project
(available in German)

State of research
(available in German)

Objective and Question
The aim of this study is to examine whether an individualized coaching has an effect on the following parameters:
- time to rehospilization and readmissionrate
- quality of life, patient activity and health literacy
- changes in health care utilization and condition related costs
- health behavior

Epidemiological, economic and health political significance
(available in German)

Relevance of objective for health care practice
(available in German)

Design and Methods

Study design and sample
The aim of this prospective study with several measuring points is to investigate chronically ill patients, selected in three groups, a) *participants of a coaching* (TN), b) patients who initially did not receive a coaching (*control group*) and c) patients who receive a coaching, but rejected (*decliners*). The data collection should be based on a written survey.
It should be collected the reason for participating the coaching, the attitude towards a coaching for health behavior problems of the control group and especially the health condition of all participants and the following dimensions:
- risk factors and clinical parameter (e.g. smoking, weight, alcohol consumption, adherence)
- psychosocial outcomes (e.g. deprssion, anxiety, symptoms)
- health beliefs, health attitudes, health behavior
- inability to work
- utilization of health care facilities, hospitalization, costs etc.
- subjectively assessed benefits of coaching
- health/treatment decisions, drug prescriptions

Measuring instruments

The instrument for the survey consists of scientifically established and validated scales as well as especially generated scales for the insured (table 1). The scales refer to mental and physical health of the participants, the general attitude to health and illness, the assessment of the coaching and changes in relation to their health behavior. It also collects routine data (type, duration and severity of illness, utilization of health care, hospital admissions, costs). Also, information to the coaching, such as frequency of contacts, duration, coaching topics and goals including prioritization were assessed.

Statement towards gender mainstreaming

(available in German)

Inclusion and exclusion criteria

Inclusion criteria:

Insurants invited to take part with chronic conditions and e.g. hospilization in the last 4-6 weeks (chronic obstructive pulmonary disease COPD, asthma, coronary artery disease, diabetes, hypertension, heart failure)

Exclusion criteria:

Insured with ongoing hospilization, hard of hearing, having no phone

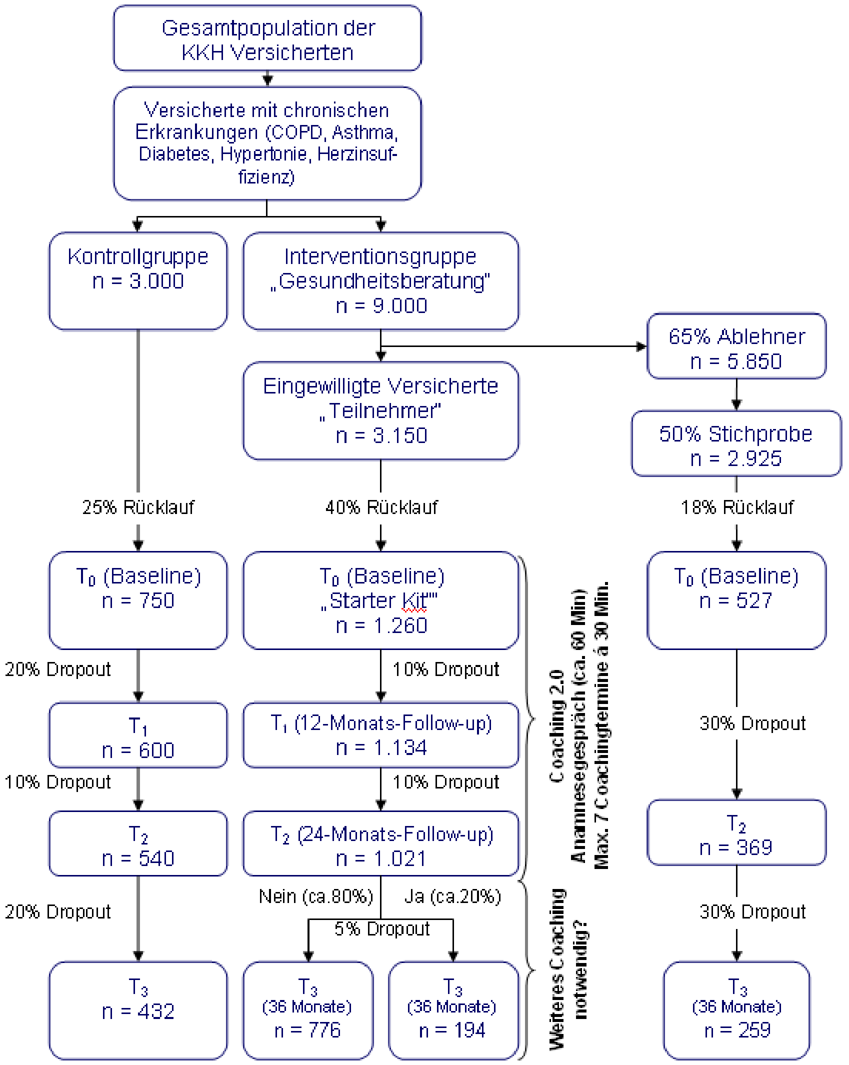

Main targets

In a randomized study, the individualized telephone coaching is evaluated for the effects compared to a control group without specific coaching.

Primary outcome:

1. Time to rehospitalization

Secondary outcomes:

2. Utilization of health care and medical costs (inpatient and outpatient)
3. Knowledge, acceptance and adherence to guideline-oriented health Recommendations (health behavior)

Supplementary questions:

4. Influence of sociodemographic variables; Influence of the diagnosis and symptoms of the disease; Influence of counseling intensity

Assumed sample size and Power calculation

In this prospective study the effectiveness and efficiency of the individualized telephone coaching, eligible insured members of the KKH Alliance for Chronic Diseases (COPD, asthma, coronary artery disease, diabetes, hypertension, heart failure) will be selected as part of a nationwide draw. A total of 12,000 insured persons are randomly assigned to the intervention group (participants in the telephone coaching) or to the control group (insured persons without telephone-based coaching) (by the KKH Allianz).
Assuming an utilization rate (enrollment to the coaching) of approximal 35% and a return rate of 40%, a sample of 1,260 insured persons (intervention group) results. Insured persons of the control group are analyzed with an assumed return rate of about 25% n = 750. From the group of "rejectors" a random sample of N = 3000 is drawn, so an assumed return rate of about 18% n = 540 insured can be included. The assumptions for the return are based on experience from the pilot study (Härter et al., 2009). In total, more than 9,000 insured persons will be contacted and around 2,500 will be included in the analysis.

Tab. 1: Operationalization of variables, data sources and time of measurement [a]

	T_0	T_1	T_2	T_3	T_4
Soziodemographic (e.g. age, gender, marital status, job-related situation)	●				
Attitude to coaching	●				
Control and causal attributions of illness and health (e.g. PAM; SOC; evtl. MHLC)	●		●		●
Symptoms / complaints acquisition / quality of life (HADS; SF-12, EQ-5D)	●	●	●	●	●
Evaluation of the coaching / benefit assessment	●	●	●	●	●
Individual risk factors / risk behaviors	●	●	●	●	●
health behavior and risk factor management	●	●	●	●	●
Participation preference in medical decisions (e.g. MSH)	●		●		●

[a] MHLC: Multidimensional Health Locus of Control Scales; (Wallston et al. 1978); HADS: Hospital Anxiety and Depression Inventory (Herrmann-Lingen, Buss and Snaith 2011) ; SF-12 = Short Form-12 Health Survey (Bullinger & Kirchberger 1998); EQ-5D = EuroQol Group 1990; PAM Patient activation measure (Hibbard 2004); SOC: stages of change (Nigg 1999)
* Especially generated scales fort he insured (s. pilotstudy)

The primary evaluations refer to the 12-month catamnesis (T_2). A 24-month follow-up (T_3) is used to check long-term effects. With regard to the 12-month follow-up (T_2), a drop-out of approximately 15% in the intervention group and approximately 30% in the control group is anticipated, so a sample of n = 1,077 insured persons in the intervention and n = 540 insured in the control group can be assumed. For the 24-month follow-up (T_3), a sam-

ple of n = 973 (about 77%) insured persons in the intervention and n = 438 insured persons in the control group (about 58%) are counted. From the group of rejectors, N = 3000 insured persons are randomly selected. With an initial response rate of approximately 18% and a dropout rate of 30% each, n = 368 patients should be analyzed for the 12-month follow-up (T_2) and n = 296 for the 24-month follow-up (T_3).
The suggested sample size is sufficient to detect even small effects with power over 90% at T_3. Thus, the study is suitable for testing the formulated hypotheses with adequate test strength and for carrying out numerous exploratory analyzes (for example in subgroups).

Statistic analysis and evaluation

- Frequencies are calculated for the evaluation of binary items and other binary outcomes. The significance check is based on chi-square tests. Adjustment for possible covariance effects is carried out by logistic regression.
- For scales and other interval-scaled outcomes, univariate variance analyzes are calculated using the study group factor (participant, control group, rejectors). Subsequent post hoc Scheffé tests were carried out for a specific intergroup comparison. Adjustment for possible covariate effects is achieved by including covariates in the statistical model (covariance analysis).
- A significance level of $p \leq .05$ is set.

Ethical giudelines and privacy

The implementation and evaluation of the project follows the principles of personal data protection. Participation in the study is generally voluntary. Users are informed about the collection and handling of the collected data. A vote of the ethics committee of the Landesärztekammer Hamburg is requested. Due to previous experience with other examinations and studies with comparable questions, no objection can be expected.

Use of research findings

Die Studie ermöglicht erstmals eine Überprüfung der Wirksamkeit sowie der gesundheitsökonomischen Relevanz dieses innovativen Gesundheitsberatungsmodells durch Krankenkassen. Die Ergebnisse können die Anwendung und Modellierung des Health Coaching-Ansatzes, den mehrere Krankenkassen derzeit verfolgen, beeinflussen.

References

1. O'Connor AM, Stacey D, Légaré F (2008). Coaching to support patients in making decisions. Brit Med J, 336(7638):228-9.
2. Härter M, Bermejo I, Schäfer L, Simon D (2009). Pilotstudie zur Evaluation der telefonischen Gesundheitsberatung der KKH. Abschlussbericht.
3. Gesundheitsberichterstattung des Bundes (2006). Gesundheit in Deutschland.

Project management

Responsible / involved persons
(available in German)

Advance performance of applicants
(available in German)

Supporting institutions/ cooperation partners
(available in German)

Quality assurance/ security
(available in German)

**Studienprotokoll für die Studie
„Evaluation eines telefonischen Gesundheitscoachings
für chronisch kranke Patienten“**

Wissenschaftliche Fragestellung

Thema des Projekts

Ein individualisiertes, telefonisches Gesundheitscoaching (GC) bei chronisch kranken Patienten wird seit 2007 von der Kaufmännischen Krankenkasse Hannover (KKH-Allianz) als zusätzliches Dienstleistungsangebot angeboten (ab 11/2009 bundesweit). Thema der Studie ist die Evaluation des GC im Hinblick auf das Gesundheitsverhalten und klinische Parameter bei den Teilnehmern. Darüber hinaus sollen die gesundheitsökonomischen Auswirkungen des GC evaluiert werden.

Stand der Forschung

Konzeptionelle Ansätze zur *Gesundheitsberatung* bzw. zum sog. *Gesundheitscoaching* (GC) wurden in den letzten Jahren entwickelt. Sie werden vor allem in telefonischer Form zur Unterstützung der Behandlung von Patienten mit chronischen Erkrankungen angeboten (O'Connor et al., 2008). Ziele eines telefonischen GC liegen primär in der *Wissensvermittlung* zu chronischen Erkrankungen und ihrer Behandlung, dem *Aufbau von Patientenkompetenzen*, der *Förderung eines konstruktiven Gesundheitsverhaltens, der Verbesserung der Arzneimittel-Adhärenz* sowie der *Verbesserung der Wahrnehmung von Gesundheitsrisiken*. Seit Juli 2007 bietet die KKH-Allianz ihren Versicherten mit chronischen, meist multimorbiden Erkrankungen ein individualisiertes telefonisches GC an, das an den *Grundsätzen der Partizipativen Entscheidungsfindung* und *Patientenorientierung* ausgerichtet ist. Durch dieses GC, das in der Regel 5-10 regelmäßige Telefonkontakte über 12-18 Monate umfasst, sollen die *Entscheidungsfindung* und ein *gesundheitsbewusstes Verhalten* durch individualisierte evidenzbasierte Informationen gefördert werden. Zentrales Anliegen ist die gemeinsame *Identifikation und Festlegung individueller Gesundheitsziele* und die *Initiierung, Begleitung und Unterstützung konkreter Verhaltensänderungen*. Eine in 2008 im Rahmen einer Evaluationsstudie (einmalige retrospektive Querschnittsbefragung bei Versicherten mit und ohne Inanspruchnahme des GC) ergab, dass die Beratungsteilnehmer vor allem mit den *vermittelten Gesundheits- bzw. Behandlungsinformationen*, den *Beratern* und dem *Beratungsprozess* sehr zufrieden waren (Härter et al. 2009). Darüber hinaus wurde von den Befragungsteilnehmern eine *subjektiv wahrgenommene Reduktion von Risikofaktoren* angegeben.

Zielsetzung und Fragestellung

Untersucht wird, ob ein individualisiertes GC Auswirkungen auf die folgenden Parameter hat:
- Zeit bis zur Wiedereinweisung ins Krankenhaus und Wiedereinweisungsrate
- Lebensqualität, Patientenaktivität und Health Literacy
- Veränderungen der Inanspruchnahme und krankheitsbedingte Kosten
- Gesundheitsverhalten

Epidemiologische, ökonomische und gesundheitspolitische Bedeutung

Die Gesundheitsversorgung in Deutschland ist mit einer stetig wachsenden Anzahl chronischer Krankheiten konfrontiert, wie Herz-Kreislauf-Erkrankungen, Erkrankungen des Muskel- und Skelettsystems, Krebserkrankungen und psychische Erkrankungen sowie Atemwegs- (z.B. COPD) und endokrinologische Erkrankungen. Die Konsequenzen dieser Entwicklung sind eine Zunahme von Arbeitsunfähigkeit, Pflegebedürftigkeit, und Frühberentungen sowie der Verlust von Lebensqualität und potentiellen Lebensjahren für die Patienten (Gesundheitsberichtserstattung des Bundes, 2006). Erschwerend kommt hinzu, dass viele Patienten nicht die für sie adäquate Behandlung erhalten bzw. aufgrund individueller Risikofaktoren/-verhaltensweisen die Behandlung nicht ausreichend effektiv ist. Insgesamt kommt es hierdurch auch zu erhöhten ökonomischen Belastungen für das Gesundheitssystem.

Relevanz der Fragestellung für die Versorgungspraxis

Patienten mit multimorbiden Problemlagen bedürfen häufig umfassender und komplexer Behandlungsmaßnahmen, die gut koordiniert sind. Beim telefonischen GC erfolgt eine individuelle, evidenzbasierte Beratung für den Umgang mit chronischen Erkrankungen; sie dient der planvollen Vorbereitung bzw. notwendigen Ergänzung einer professionellen (fach)ärztlichen Versorgung (O'Connor et al., 2008). Gesundheitscoaching wird seit einigen Jahren von zahlreichen Krankenkassen mittels spezifisch ausgebildeter Fachkräfte angeboten. Es handelt es sich um einen für das deutsche Gesundheitswesen neuen und innovativen Ansatz, der allerdings unzureichend evaluiert ist. Aus den bisher vorliegenden Studien (Härter et al. 2009) können keine kausalen Schlüsse hinsichtlich der Effektivität eines derartigen Coachingangebots gezogen werden. Für die Ermittlung von aussagekräftigen Ergebnissen zur Effektivität bedarf es *prospektiv angelegter, kontrollierter Längsschnittstudien*, in der Teilnehmer bzw. Nicht-Teilnehmer *vor*, *während* und *im Anschluss an die Beratung* über einen längeren Zeitraum befragt werden, um mittel- und langfristige Effekte evaluieren zu können.

Design und methodische Vorgehensweise

Studienphasen und Datenerhebung

Im Rahmen einer prospektiven Studie mit mehreren Messzeitpunkten sollen chronisch kranke Patienten in drei Gruppen, a) *Teilnehmer der Beratung (TN)*, b) Personen, die das Angebot zur Teilnahme zunächst nicht erhalten haben (*Kontrollgruppe*) und c) Personen, die das Beratungsangebot erhalten, aber abgelehnt haben (*Ablehner*) untersucht werden (s. Abb. 1). Die Datenerhebung soll anhand einer schriftlichen Befragung erfolgen. Hierbei soll neben der Erfassung der Gründe für die Teilnahme an der Beratung bei den Beratungsteilnehmern bzw. der Einstellung zu einer Beratung bei Gesundheitsproblemen bei der Kontrollgruppe v.a. das gesundheitliche Befinden aller Studienteilnehmer sowie folgende zentrale Dimensionen erfasst werden, insbesondere:

- Risikofaktoren und klinische Parameter (z.B. Rauchen, Gewicht, Alkohol, Adhärenz);
- Psychosoziale Belastungen (z.B. Depression, Angst, Beschwerden);
- Health Beliefs, gesundheitliche Einstellungen, Gesundheitsverhalten;
- Arbeitsunfähigkeit
- Inanspruchnahme von Versorgungseinrichtungen, Krankenhausaufenthalte, Kosten etc.;
- Subjektiv eingeschätzter Nutzen der Beratung;
- Gesundheits- bzw. Behandlungsentscheidungen, Arzneimittelverordnungen.

Messinstrumente

Das Instrumentenset für die Versichertenbefragung besteht sowohl aus wissenschaftlich etablierten und validierten Skalen als auch aus speziell für die Befragung der Versicherten generierten Fragebogen-Skalen (s. Tab. 1). Die Skalen beziehen sich auf die psychische und physische Befindlichkeit der Befragten, die allgemeine Einstellung der Versicherten zu Gesundheit und Krankheit, die Einschätzung des GC sowie Veränderungen der Versicherten im Bezug auf ihr Gesundheitsverhalten (inkl. klinischer Parameter). Ferner werden Routine-Versorgungsdaten (Art, Dauer und Schweregrad der Erkrankung; Inanspruchnahme von Gesundheitsdienstleistungen, Krankenhauseinweisungen, Kosten) erhoben. Ergänzt werden diese Daten durch Angaben zum GC, wie z.B. Häufigkeit der Kontakte, Dauer, Beratungsthemen und -ziele inkl. Priorisierung.

Aussagen zum Gender Mainstreaming

Bei der Ausgestaltung der Inhalte und Ziele sowie bei der Evaluation inklusive der Umsetzung des GC werden die spezifischen Bedürfnisse und Lebensrealitäten von Frauen und Männern explizit einbezogen, auch wird Rücksicht auf migrantenrelevante Bedürfnislagen genommen.

Einschluss- und Ausschlusskriterien

Einschlusskriterien:

Zum GC eingeladene Versicherte mit chronischen Erkrankungen und z.B. Krankenhausaufenthalt in den letzten 4-6 Wochen (COPD, Asthma, KHK, Diabetes, Hypertonie, Herzinsuffizienz)

Ausschlusskriterien:

Versicherte mit laufendem Krankenhausaufenthalt, Schwerhörigkeit, nicht im Besitz eines Telefons.

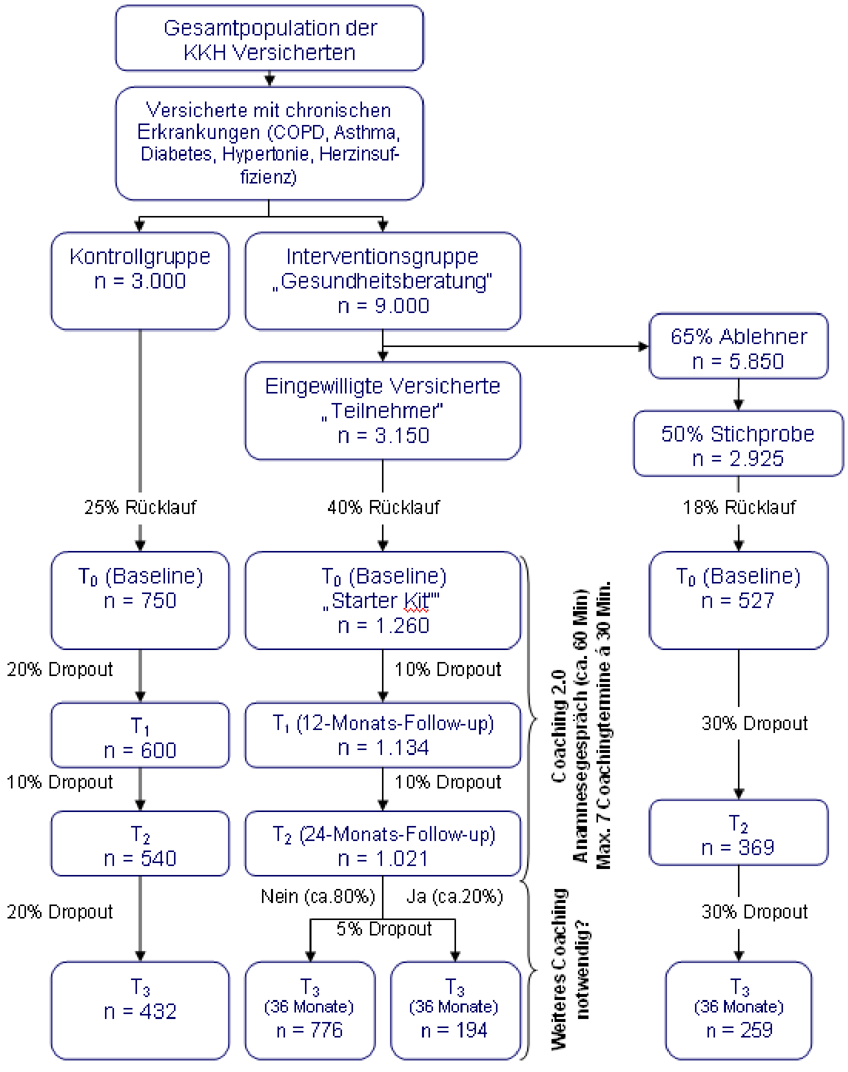

Abb. 1: Studiendesign und Übersicht über die Studienphasen

Hauptzielgrößen

In einer randomisierten Studie wird das individualisierte telefonische GC bezüglich der Auswirkungen im Vergleich zu einer Kontrollgruppe ohne spezifisches Coaching evaluiert.

Primäre Zielgröße:

1. Zeit bis zur Wiedereinweisung ins Krankenhaus

Sekundäre Zielgrößen:

2. Inanspruchnahmeverhalten und Krankheitskosten (stationär und ambulant)
3. Wissen, Akzeptanz und Adhärenz bzgl. leitlinienorientierter Gesundheitsempfehlungen (Gesundheitsverhalten)

Ergänzende Fragestellungen

4. Einfluss soziodemographischer Variablen; Einfluss der Diagnose und Symptomatik der Erkrankung; Einfluss der Beratungsintensität

Vorgeschlagene Stichprobengröße und Powerkalkulation

Für die prospektive Studie zur Effektivität und Effizienz des individualisierten telefonischen GC werden im Rahmen einer bundesweiten Ziehung in Frage kommende Versicherte der KKH-Allianz mit chronischen Erkrankungen (COPD, Asthma, KHK, Diabetes, Hypertonie, Herzinsuffizienz) ausgewählt. Ingesamt werden *12.000 Versicherte zur Interventionsgruppe* (Teilnehmer am telefonischen GC) bzw. zur Kontrollgruppe (Versicherte ohne GC) (durch die KKH Allianz). Unter Annahme einer Inanspruchnahmequote (Einschreibung zum GC) von ca. 35% und einer Rücklaufquote von 40% ergibt sich eine Stichprobe von *1.260 Versicherten (Interventionsgruppe)*. Aus den Versicherten der *Kontrollgruppe* werden bei einer angenommenen Rücklaufquote von ca. 25% *n=750 Versicherte* analysiert. Aus der Gruppe der „Ablehner“ wird eine zufällige Stichprobe von N=3000 gezogen, so dass bei einer angenommenen Rücklaufquote von ca. 18% *n= 540* Versicherte einbezogen werden können. Die Annahmen zum Rücklauf basieren auf Erfahrungswerten aus der Pilotstudie zum GC (Härter et al. 2009). Insgesamt werden über *9.000 Versicherte a*ngeschrieben und ca. *2.500* in die Analysen einbezogen.

Tab. 1: Operationalisierung der Variablen, Datenquellen und Messzeitpunkte[a]

	T_0	T_1	T_2	T_3	T_4
Soziodemographie (z.B. Alter, Geschlecht, Familienstand, berufliche Situation)	●				
Einstellung zum GC	●				
Kontroll- und Kausalattributionen bzgl. Krankheit und Gesundheit (z.B. PAM; SOC; evtl. MHLC)	●		●		●
Symptom-/Beschwerdenerfassung/Lebensqualität (HADS; SF-12, EQ-5D)	●	●	●	●	●
Bewertung des GC / Nutzenbewertung	●	●	●	●	●
Individuelle Risikofaktoren / Risikoverhaltensweisen*	●	●	●	●	●
Gesundheitsverhalten und Umgang mit Risikofaktoren*	●	●	●	●	●
Beteiligungspräferenz bei medizinischen Entscheidungen (z.B. MSH)	●		●		●

[a] MHLC: Multidimensional Health Locus of Control Scales; (Wallston et al. 1978); HADS: Hospital Anxiety and Depression Inventory (Herrmann-Lingen, Buss and Snaith 2011) ; SF-12 = Short Form-12 Health Survey (Bullinger & Kirchberger 1998); EQ-5D = EuroQol Group 1990; PAM Patient activation measure (Hibbard 2004); SOC: stages of change across ten health behaviors (Nigg 1999).

* Spezifisch für die Befragung der Versicherten generierte Skalen (s. Pilotstudie)

Die primären Auswertungen beziehen sich auf die 12-Monats-Katamnese (T_2). Eine 24-Monats-Katamnese (T_3) wird genutzt, um langfristige Effekte zu überprüfen. Hinsichtlich der 12-Monats-Katamnese (T_2) wird mit einem Drop-out von ca. 15% in der Interventionsgruppe und ca. 30% in der Kontrollgruppe gerechnet, so dass von einer Stichprobe von n=1.077 Versicherte in der Interventions- und n=540 Versicherte in der Kontrollgruppe ausgegangen werden kann. Für die 24-Monats-Katamnese (T_3) wird mit einer Stichprobe von n= 973 (ca. 77%) Versicherte in der Interventions- und n= 438 Versicherte in der Kontrollgruppe (ca. 58%) gerechnet.
Aus der Gruppe der Ablehner werden zufällig N= 3000 Versicherte ausgewählt. Bei einer initialen Rücklaufquote von ca. 18% und einer Dropout-Rate von jeweils 30% sollen zur 12-Monats-Katamnese (T_2) n=368 und zur 24-Monats-Katamnese (T_3) n = 296 Ablehner analysiert werden.
Die vorgeschlagene Stichprobengröße ist ausreichend, um sogar bei T_3 kleine Effekte mit einem Power über 90% zu entdecken. Damit ist die Studie geeignet um die formulierten Hypothesen mit einer angemessenen Teststärke zu prüfen und die Durchführung von zahlreichen explorativen Analysen (z.B. in Subgruppen) zu ermöglichen.

Statistische Analysen und Auswertung

- Für die Auswertung von binären Einzelitems sowie weiteren binären Outcomes werden Häufigkeiten berechnet. Die Signifikanzprüfung erfolgt anhand von Chi-Quadrat-Tests. Adjustierung für mögliche Kovariaten-Effekte erfolgt mittels logistischer Regression.
- Bei Skalen und weiteren intervallskalierten Outcomes werden univariate Varianzanalysen mit dem Faktor Studiengruppe (Teilnehmer, Kontrollgruppe, Ablehner) berechnet. Für einen spezifischen Intergruppenvergleich wurden im Anschluss post hoc-Scheffé-Tests durchgeführt. Adjustierung für mögliche Kovariaten-Effekte erfolgt durch den Einschluss von Kovariaten in das statistische Modell (Kovarianzanalyse).
- Ein Signifikanzniveau von $p \leq .05$ wird festgelegt.

Forschungsethik und Datenschutz

Die Durchführung und Auswertung des Vorhabens folgt den Grundsätzen des persönlichen Datenschutzes. Die Teilnahme an der Studie ist grundsätzlich freiwillig. Die Nutzer werden über die Erhebung und den Umgang mit den erhobenen Daten informiert. Ein Votum der Ethikkommission der Landesärztekammer Hamburg wird beantragt. Aufgrund der Vorerfahrungen mit anderen Untersuchungen und Studien mit vergleichbaren Fragestellungen ist nicht mit einer Beanstandung zu rechnen.

Nutzen und Verwendungsmöglichkeit der Forschungsergebnisse

Die Studie ermöglicht erstmals eine Überprüfung der Wirksamkeit sowie der gesundheitsökonomischen Relevanz dieses innovativen Gesundheitsberatungsmodells durch Krankenkassen. Die Ergebnisse können die Anwendung und Modellierung des Health Coaching-Ansatzes, den mehrere Krankenkassen derzeit verfolgen, beeinflussen.

Literatur

4. O’Connor AM, Stacey D, Légaré F (2008). Coaching to support patients in making decisions. Brit Med J, 336(7638):228-9.
5. Härter M, Bermejo I, Schäfer L, Simon D (2009). Pilotstudie zur Evaluation der telefonischen Gesundheitsberatung der KKH. Abschlussbericht.

6. Gesundheitsberichterstattung des Bundes (2006). Gesundheit in Deutschland.

Projektmanagement

Verantwortliche/Beteiligte

Name	Institut	Telefon, Fax, E-mail	Verantwortlichkeit
Prof. Dr. Dr. M. Härter	Universitätsklinikum Hamburg-Eppendorf; Institut für Medizinische Psychologie	Tel.: 040 / 7410-52978; Fax: 040 / 7410-54965 m.haerter@uke.uni-hamburg.de	Projektleitung
Dr. I. Bermejo	Universitätsklinikum Freiburg, Abt. Psychiatrie und Psychotherapie	Tel.: 0761 / 2706982; Fax: 0761 / 270-6989 isaac.bermejo@uniklinik-freiburg.de	Projektberatung
Dr. L. Kriston	Universitätsklinikum Hamburg-Eppendorf; Institut für Medizinische Psychologie	Tel: 040 / 7410-56849; Fax: 040 / 7410-54965 l.kriston@uke.uni-hamburg.de	Biometrie
Prof. Dr. H.-H. König, MPH	Universitätsklinikum Leipzig Professur für Gesundheitsökonomie	Tel: 0341 / 972-4561; Fax: 0341 / 972-4569 hans-helmut.koenig@medizin.uni-leipzig.de	Gesundheitsökonomie
Dipl.Psych. S.Dwinger	Universitätsklinikum Hamburg-Eppendorf; Institut für Medizinische Psychologie	Tel: 040 / 7410-56204; Fax: 040 / 7410-54965 s.dwinger@uke.uni-hamburg.de	Projektmitarbeiterin

Vorleistungen beteiligter Wissenschafter

Der Antragsteller arbeitet seit Jahren intensiv am Thema der Patientenorientierung mit unterschiedlichen Schwerpunkten; er hat jahrelange und vielfältige Erfahrungen in klinischen und methodischen Projekten zur Patientenorientierung und -beteiligung. Aktuell werden am Institut für Medizinische Psychologie am UKE in Hamburg im Rahmen des Förderschwerpunkts „Chronische Krankheiten und Patientenorientierung“ mehrere Projekte (+ AG Kommunikation/Networking) durchgeführt.

Härter, M., Barth, J., & Friderich, C., Wagensommer, C. & Koch, U. (1994). Participation in and effects of health counseling for the health promotion of musculoskeletal disorders. Patient Education and Counseling, 25, 121-130.

Härter, M., Barth, J., & Friderich, C., Wagensommer, C. & Koch, U. (1996). Inanspruchnahme und Effekte von Gesundheitsberatungen zur Gesundheitsförderung bei Erkrankungen am Stütz- und Bewegungsapparat. Prävention, 19 (1), 10-13.

Härter, M., Battlehner, J., Münscher, A., Graul, J. & Maurischat, C. (2005). Erfassung der Veränderungsmotivation bei Tinnituspatienten - eine Studie zum Transtheoretischen Modell. HNO, 53, 707-715.

Härter, M., Loh, A. & Spies, C. (Hrsg.) (2005). Gemeinsam entscheiden erfolgreich behandeln – Neue Wege für Ärzte und Patienten im Gesundheitswesen. 247 Seiten. Köln: Deutscher Ärzte-Verlag.

Loh, A., Simon, D., Kriston, L. & Härter, M. (2007). Shared Decision-Making in Medicine. Deutsches Ärzteblatt, 104 (21), A1483-1488.

Unterstützende Einrichtungen/ Kooperationspartner

Name	Institut	Telefon, Fax, E-mail	Verantwortlichkeit/Rolle
Dr. Lutz Herbarth	KKH - Allianz / Individuelles Gesundheitsmanagement - Hauptverwaltung	0511 2802 3680; lutz.herbarth@kkh-allianz.de	Koordination der Zusammenarbeit mit den Beratungszentren, Zusammenstellung der Versichertendaten für Befragungen und gesundheitsökonomische Analyse
Fr. Gisela Bräuer	Versorgungszentrum (VZ) München	089 53298-9001; gisela.braeuer@kkh-allianz.de	Koordination der Befragung im Versorgungsbereich München
Fr. Bianka Doberenz	Versorgungszentrum (VZ) Halle	0345 685880 5001; bianka.doberenz@kkh-allianz.de	Koordination der Befragung im Versorgungsbereich Halle

Qualitätssicherung/ Sicherheit

Die Gewährleistung der Datensicherheit erfolgt in enger Kooperation mit dem Datenschutzbeauftragten des Universitätsklinikums Hamburg-Eppendorf und der entsprechenden Einrichtung des kooperierenden Leistungsträgers (KKH-Allianz). Als Qualitätsanforderungen gelten die Richtlinien, die von der Gesellschaft für Versicherungswissenschaft und –gestaltung e.V. (GVG) erstellt wurden. Die Befragung und die Auswertung der Daten erfolgte nach wissenschaftlichen Standards, bei denen die Antworten der Teilnehmer/innen ausschließlich pseudonymisiert ausgewertet wurden. Die Angaben der Befragung wurden mit einzelnen Krankheits- und Behandlungsdaten der KKH-Versicherten verknüpft (Bundesland, Intensität der Beratung, Anzahl der Kontakte und Indikation), aber nur gruppenweise ausgewertet, so dass keine Rückschlüsse auf einzelne Versicherte möglich waren. Sowohl das Studiendesign als auch das Vorgehen bei der Befragung, der Umgang mit den Versichertendaten und die Auswertungsstrategie wurden mit der Abteilung Datenschutz der KKH abgestimmt und von der Ethikkommission der Universität Hamburg beraten.
