# Supplementary material for: Effects of telephone-based health coaching on patient-reported outcomes and health behavior change: A randomized controlled trial
Source: PLoS One. 2020 Sep 22;15(9):e0236861. doi: 10.1371/journal.pone.0236861 (PMC7508388; doi:10.1371/journal.pone.0236861)
Supplement: S3 File — (PDF) [file pone.0236861.s009.pdf]

Ärztekammer Hamburg · Postfach 76 01 09 · 22051 Hamburg

ETHIK-KOMMISSION DER
ÄRZTEKAMMER
HAMBURG
Körperschaft des öffentlichen Rechts

Herrn
Prof. Dr. med. Dr. phil. Härter
Institut und Poliklinik für Medizinische Psychologie
Universitätsklinikum Hamburg Eppendorf
Martinistr. 52
20246 Hamburg

12.05.2011

Bearb.-Nr.: **PV3567 (Bitte stets angeben!)**
Studie: „Effektivität und Effizienz eines individualisierten telefonischen Gesundheitscoaching bei chronischen Erkrankungen"

Sehr geehrter Herr Kollege Härter,

über Ihr oben bezeichnetes, zur Primärberatung vorgelegtes Projekt hat die Ethik-Kommission ausführlich beraten.

Das Vorhaben entspricht den berufsrechtlichen bzw. gesetzlichen Anforderungen. Die Ethik-Kommission stimmt dem Vorhaben zu.

Die Kommission weist darauf hin, dass die Verantwortung des Versuchsleiters für das Forschungsvorhaben und seine Durchführung durch das obige Votum der Kommission nicht berührt wird.

Sie werden gebeten, die Ethik-Kommission über alle schwerwiegenden oder unerwarteten Ereignisse, die während der Studie auftreten und die die Sicherheit der Studienteilnehmer gefährden, in Verbindung mit Ihrer Stellungnahme zu unterrichten.
Die Kommission geht davon aus, dass die personenbezogenen Daten der Probanden/ Patienten den datenschutzrechtlichen Vorschriften entsprechend behandelt werden.

Die Ethik-Kommission erwartet, dass ihr nach Abschluss des Projektes unaufgefordert ein Abschluss-Bericht übersandt wird (unter Angabe der Bearb.-Nr.), aus dem der Erfolg/Misserfolg der Studie sowie Angaben darüber, ob die Studie abgebrochen oder geändert bzw. ob Regressansprüche geltend gemacht wurden, ersichtlich sind.

Mit verbindlicher Empfehlung
Im Auftrage der Kommission:

Prof. Dr. med. Th. Weber
- Vorsitzender -

P.S. Die Ethik-Kommission arbeitet auf der Grundlage deutschen Rechts und Berufsrechts sowie in Anlehnung an die ICH-GCP

Humboldtstraße 67a · 22083 Hamburg
Telefon 040 / 20 22 99-240 · Fax 040 / 20 22 99-410
ethik@aekhh.de · www.aerztekammer-hamburg.de
Geschäftsführung: Dr. Silke Schrum

Bankverbindung:
Deutsche Apoth. u. Ärztebank, BLZ 300 606 01, Konto-Nr. 000 1346 113
BIC DAAEDEDD, IBAN DE71 3006 0601 000 1346 113
