# Supplementary figures and images for: Effects of telephone-based health coaching on patient-reported outcomes and health behavior change: A randomized controlled trial
Source: PLoS One. 2020 Sep 22;15(9):e0236861. doi: 10.1371/journal.pone.0236861 (PMC7508388; doi:10.1371/journal.pone.0236861)

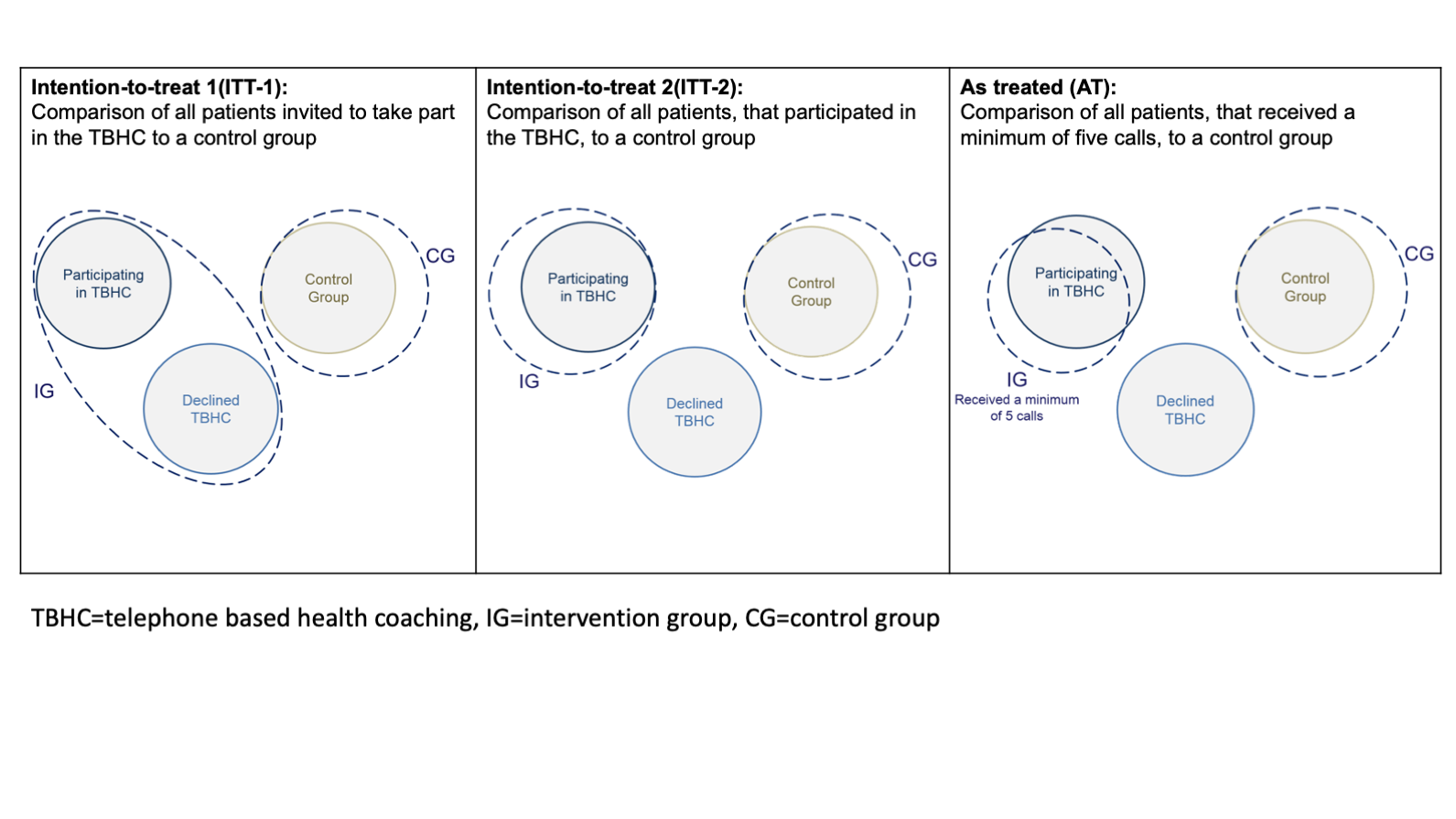

Supplement: S1 Fig — (TIFF) [file pone.0236861.s010.tiff]
